# SEPAR enables spatial metagene discovery and associated molecular pattern characterization in spatial transcriptomics and multi-omics datasets

**DOI:** 10.1038/s42003-025-09340-w

**Published:** 2025-12-10

**Authors:** Lei Zhang, Ying Zhu, Shuqin Zhang

**Affiliations:** 1 School of Mathematical Sciences, Fudan University, Shanghai, China; 2 State Key Laboratory of Brain Function and Disorders, MOE Frontiers Center for Brain Science, Institutes of Brain Science and Department of Neurosurgery, Huashan Hospital, Fudan University, Shanghai, China; 3 Center for Applied Mathematics, Research Institute of Intelligent Complex Systems, and Shanghai Key Laboratory for Contemporary Applied Mathematics, Fudan University, Shanghai, China

**Keywords:** Computational models, Data mining, Software

## Abstract

Spatially resolved transcriptomics (SRT) profiles gene expressions at near- or sub-cellular resolution while preserving their spatial context, yet interpreting SRT data to understand spatial cellular and molecular organization remains challenging. Most existing computational methods focus on global spatial domains but overlook localized structures driven by specific gene subsets. Here, we introduce SEPAR, an unsupervised framework that leverages spatial metagenes to analyze SRT data by integrating gene activity and spatial neighborhood relationships. It enables multiple downstream analyses including: identifying metagene pattern-specific genes, detecting spatially variable genes (SVGs), delineating spatial domains, and refining expression signals. Evaluated on diverse datasets, SEPAR reveals biologically meaningful gene ontologies and cell types in gene sets linked to metagene patterns, identifies SVGs with higher accuracy, and enhances biological signals with gene refinement. In spatial multi-omics data, it uncovers co-localized molecule correlations in spatial CITE-seq and coordinated gene-peak relationships in MISAR-seq, offering insights into spatial molecular interactions.

## Abstract (web summary)

SEPAR is a computational approach that uses spatial metagenes to reveal localized gene programs, identify spatially variable genes, delineate spatial domains, and refine gene expressions for spatial transcriptomics, and uncover coordinated interactions for spatial multi-omics.

## Introduction

Spatially resolved transcriptomics (SRT) technologies, ranging from spot-based capture techniques to high-resolution imaging methods such as 10 × Visium^1,2^, Slide-seq^3,4^ and MERFISH^5^, have revolutionized biomedical research by providing gene expression levels at near- or sub-cellular resolution while preserving their precise spatial localizations. This advancement enables researchers to investigate the spatial organization of various cell types, explore cellular functions and cell-cell interactions, as well as examine spatially distinct gene expression patterns^6–10^. Such insights contribute to a more comprehensive understanding of complex biological systems and tissue architecture. Leveraging the success of SRT technologies, spatial molecular profiling has evolved to simultaneously capture multiple molecular modalities recently. For example, spatial CITE-seq^11^ combines transcriptome profiling with protein abundance measurement, while MISAR-seq^12^ facilitates concurrent profiling of gene expression and chromatin accessibility (ATAC) with spatial resolution. These multi-omics measurements facilitate studies of the interplay between transcriptional regulation, protein expression, and chromatin states, offering deeper insights into cellular function and tissue organization.

Various methods have been developed to identify spatial domains with similar gene expressions for SRT data, such as BayesSpace^13^, STAGATE^14^, GraphST^15^, BASS^16^, SpaGCN^17^, spaVAE^18^, and so on. While these methods excel in capturing global tissue architectures, they have serious limitations in resolving fine-grained spatial structures inherent in the data. A key issue is their focus on dominant expression patterns shared across all genes, overlooking localized patterns driven by specific gene subsets that may define functionally specialized niches or rare cell states^19^.

To address these limitations, we adopt the concept of metagenes, which has been proven successful in traditional transcriptomics analysis^20,21^. Metagenes, as weighted linear combinations of functionally related genes, represent coordinated transcriptional programs that can recover underlying biological processes or key phenotypes^21^. Samples that exhibit similar expression patterns in specific gene subsets can be extracted by summarizing their gene expression in terms of metagene expression patterns. This process can be achieved using matrix factorization methods, particularly Non-negative Matrix Factorization (NMF), which has been proven effective in extracting interpretable biological features^22^.

Applications of NMF-based methods to single-cell RNA sequencing data have demonstrated its capability in revealing coordinated gene expression programs, but these methods do not incorporate spatial information. To bridge this gap, various spatial-aware approaches have emerged. SpiceMix^23^ employs a probabilistic integration of non-negative matrix factorization with hidden Markov random fields (NMF-HMRF) to jointly infer metagenes and their spatial affinities from cellular neighborhood graphs. Nonnegative spatial factorization (NSF)^24^ employs Gaussian process priors with nonnegative constraints to model global spatial dependencies, and its hybrid version NSFH further incorporates non-spatial components for residual variation modeling. While these NMF-based spatial methods provide interpretable metagene representations, they face computational scalability challenges when applied to large datasets. Alternative frameworks integrate spatial context through different paradigms: GraphPCA^25^ incorporates spatial graphs into principal components analysis, STAMP^26^ leverages graph convolutional networks with deep generative models, and Hotspot^27^ identifies spatially autocorrelated gene modules without matrix factorization. Although these methods offer distinct analytical perspectives, they utilize alternative computational paradigms rather than matrix factorization that directly yields interpretable metagene patterns.

Identifying spatially variable genes (SVGs) that exhibit distinct spatial expression patterns is also crucial in SRT analysis. Different from pattern-specific genes that show notable signals on particular metagene expression patterns, each SVG may cover one or more spatial patterns. By identifying SVGs, researchers can explore the relationship between spatial organization and molecular cell function^28^. Furthermore, SVGs enhance spatially-aware clustering and trajectory inference, contributing to better understanding of tissue morphology and inter-cellular communication^13,17,29–31^. Current methods for identifying SVGs include SpatialDE^10^, SPARK^32^, SPARK-X^33^, STAMarker^34^, and so on. SPARK, SPARK-X, and SpatialDE utilize various statistical frameworks, such as Gaussian process regression and nonparametric approaches, to test spatial variance and dependence of gene expression. However, these methods conduct hypothesis tests independently for each gene and fail to determine their corresponding spatial patterns. STAMarker, a model based on graph neural networks, relies on a pre-trained clustering model, making it difficult to identify genes that do not contribute to clustering.

For spatial multi-omics integration, several specialized methods have emerged. SpatialGlue^35^ provides a graph-based framework for integrating spatial transcriptomics with other omics data using dual attention mechanisms. MISO^36^ offers multimodal feature extraction and spatial clustering for integrating diverse omics modalities with tissue histology data. SpaMultiVAE^18^ extends variational autoencoders for joint modeling of spatial RNA-protein data by incorporating protein background/foreground mixture distributions. CellPie^37^ uses joint NMF to integrate gene expression with histopathological image features for spatial factor discovery at high resolution. MEFISTO^38^ employs Gaussian process-based factor analysis for identifying temporal and spatial patterns across modalities. However, these methods face challenges in handling modality-specific variations in scales, noise levels, and feature dimensions, which can compromise cross-modality correlation discovery and spatial pattern identification.

To address the above challenges in SRT data analysis, we developed a Spatial metagene Expression PAttern Recognition (SEPAR) method based on graph-regularized non-negative matrix factorization^39^ to identify distinct spatial expression patterns of metagenes. SEPAR integrates gene expression and spatial coordinates, capturing complementary spatial expression patterns across genes while ensuring computational efficiency. It offers several key advantages. First, SEPAR fully leverages the interpretability of NMF, extracting spatial expression patterns of metagenes^21^ and pattern-specific genes, facilitating enrichment analysis. Second, SEPAR efficiently handles various downstream tasks such as SVG identification, unsupervised spatial clustering, and gene refinement. Third, SEPAR does not rely on prior assumptions on data distribution, allowing seamless extension to multimodal spatial data analysis. Last, SEPAR can be extended to multislice version, enabling simultaneous analysis of adjacent tissue sections. We evaluated SEPAR across various datasets, including SRT data from 10 × Genomics Visium^1,2^, Stereo-seq^40^, MERFISH^5^, and osmFISH^41^, as well as multi-omics data from spatial CITE-seq^11^ and MISAR-seq^12^. We benchmarked SEPAR against state-of-the-art methods for different tasks. SEPAR consistently delivered robust analytical outcomes.

## Results

### Overview of SEPAR

**Fig. 1:**
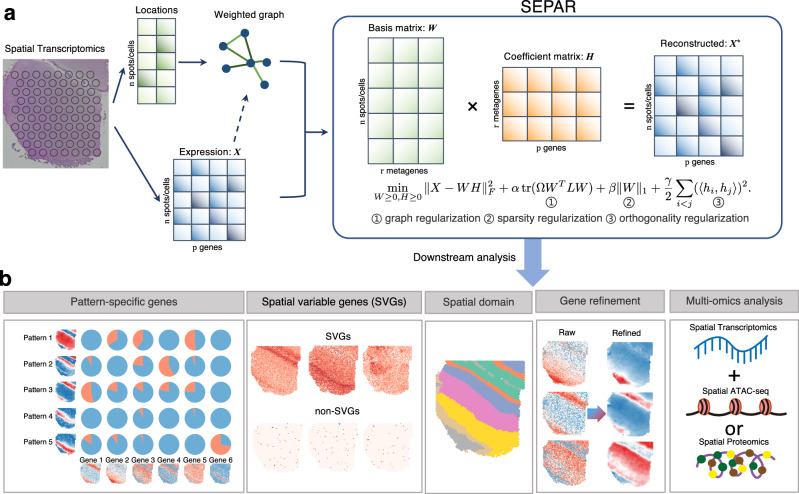
Overview of SEPAR framework and its applications. **a** SEPAR is a framework based on graph-regularized NMF designed to identify spatially aware metagene patterns using both spatial location and gene expression as input. Spatial location data is leveraged to construct weighted graph regularization capturing the spatial relationships of the spots or cells. Sparsity regularization and dissimilarity regularization are applied to ensure distinct spatial metagene patterns. **b** SEPAR supports efficient and robust downstream analyses of SRT data, including metagene expression pattern recognition, pattern-specific gene analysis, SVG identification, spatial domain delineation, gene expression denoising and spatial multi-omics data analysis.

SEPAR is a framework based on graph-regularized non-negative matrix factorization^39^, specifically designed to identify the underlying spatial metagenes and their expression patterns in SRT data (Fig. 1, “Methods”). The gene expression matrix is modeled as weighted combinations of metagenes, regularized by the terms that account for the spatial relationships of the spots and that enforce the metagene dissimilarity, ensuring clear and distinct patterns. The SEPAR framework is formulated as the following optimization problem:

$$\mathop{\min }\limits_{W\ge 0,H\ge 0}\parallel X-WH{\parallel }_{F}^{2}+\alpha {{\rm{tr}}}\,(\Omega {W}^{T}LW)+\beta \parallel W{\parallel }_{1}+\frac{\gamma }{2} \mathop{\sum }\limits_{i < j}{(\langle {h}_{i},{h}_{j}\rangle )}^{2}.$$

In this formulation, *X*_*n*×*p*_ denotes the gene expression matrix of *n* spots (or cells) and *p* genes. Matrix *W*_*n*×*r*_ represents the *r* underlying spatial metagene expression patterns in all the spots (or cells). Matrix *H* has size *r* × *p*, where each row *h*_*i*_ (*i* = 1, …, *r*) defines the expression profile of the *i*-th metagene across the genes. The spatial relationships of spots/cells are incorporated through the graph Laplacian term ${{\rm{tr}}}\,(\Omega {W}^{T}LW)$, where *L* represents the graph Laplacian matrix and *Ω* denotes pattern importance weights. The sparsity regularization ∥*W*∥_1_ mitigates noise effects in the spatial patterns. The orthogonality term ${\sum }_{i < j}{(\langle {h}_{i},{h}_{j}\rangle )}^{2}$ ensures distinct metagenes by penalizing similarities between different metagene signatures. The hyperparameters *α*, *β*, and *γ* control the relative strengths of spatial regularization, sparsity enforcement, and metagene orthogonality, respectively. This framework is also extended to multislice scenarios (**Methods**).

The results derived from SEPAR enable various downstream analyses, including spatial expression pattern recognition of metagenes, pattern-specific gene analysis, SVG identification, spatial domain delineation, gene expression denoising, and the analysis of spatial multi-omics data. The columns of matrix *W* represent the distinct spatial expression patterns of the metagenes. Pattern-specific genes, which are highly expressed in particular spatial regions, are pinpointed by measuring the similarities between the given gene expression profiles and the underlying spatial metagene expression patterns. As SVGs can be expressed across multiple spatial metagene patterns, they are identified by evaluating the distance between each gene and combinations of the spatial patterns. Additionally, the decomposed patterns facilitate spatial domain identification by directly applying *k*-means to matrix *W*. The denoised gene expression matrix is reconstructed by combining *W* and *H*. By horizontally concatenating the spatial multi-omics data into a larger matrix *X*, SEPAR can be directly applied to infer the underlying structures and associated molecular signatures.

We comprehensively evaluated the performance of SEPAR across diverse spatial transcriptomics datasets and multiple scenarios. We first demonstrated SEPAR’s capabilities in the DLPFC dataset generated using 10 × Visium^1^ and the mouse olfactory bulb dataset generated by Stereo-seq^40^, where it effectively identified biologically meaningful spatial metagene patterns and SVGs. For image-based spatial transcriptomics, we validated the performance of SEPAR using datasets generated by MERFISH^42^ and osmFISH^41^, focusing on spatial domain identification and gene expression refinement. Furthermore, we extended SEPAR’s application to multi-omics scenarios, demonstrating its versatility with the datasets generated by spatial CITE-seq^11^ and MISAR-seq^12^, highlighting its robustness in managing complex spatial molecular data. In addition, SEPAR can be extended to handle multislice SRT data, which was demonstrated with the DLPFC dataset^1^. Beyond well-structured tissues, we also validated SEPAR’s performance in disease contexts using colorectal cancer VisiumHD data^43^ (Supplementary Note 1). These extensive experiments illustrate that SEPAR not only achieves superior or competitive performance compared to existing methods but also offers computational efficiency and broad applicability across different spatial molecular profiling platforms.

### SEPAR reveals layer-specific patterns and robust SVGs in human dorsolateral prefrontal cortex

**Fig. 2:**
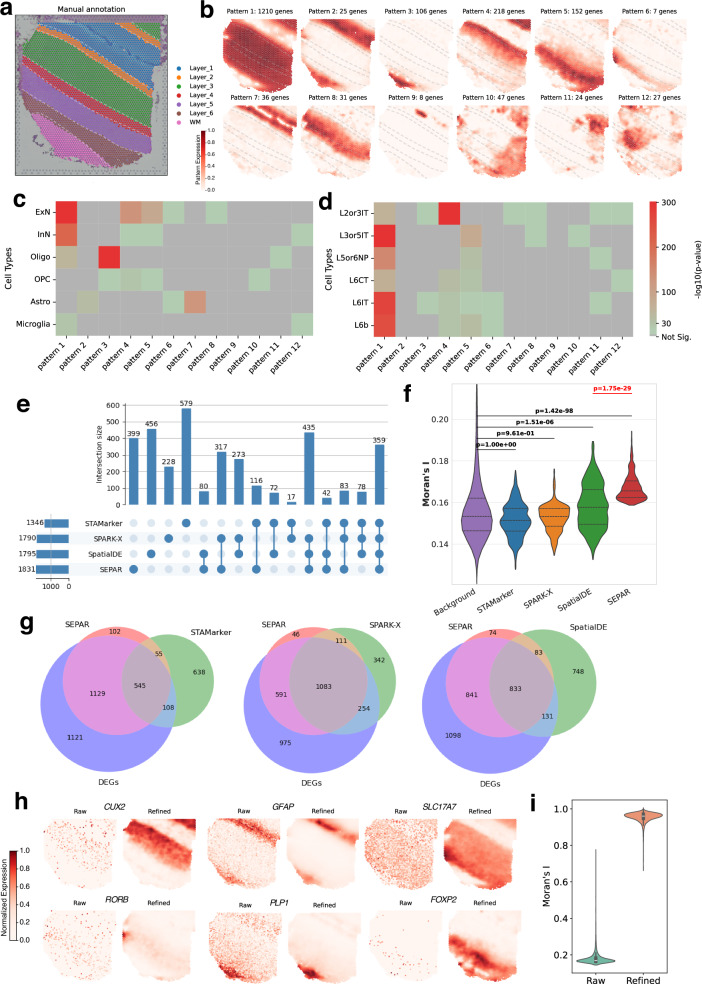
Analysis of section 151507 of DLPFC dataset^1^ generated by 10 × Visium using SEPAR. **a** Manual annotations of section 151507 from original research^1^. **b** 12 spatial metagene patterns recognized by SEPAR with number of pattern-specific genes. **c** Cell-type enrichment analysis on the gene sets corresponding to each pattern by CellGO^44^, using major brain cell types (ExN, InN, Oligo, OPC, Astro, Microglia). **d** Cell-type enrichment analysis for excitatory neuron (ExN) subtypes (L2/3IT, L3/5IT, L5/6NP, L6CT, L6IT, L6b). **e** UpSet plot for SVGs identified by STAMarker, SPARK-X, SpatialDE and SEPAR. **f** Comparison of Moran’s I values for uniquely identified SVGs by each method. The sample sizes of background, STAMarker, SPARK-X, SpatialDE and SEPAR are 8,026; 463; 227; 270; and 399, respectively. Background genes are all genes excluding the common SVGs identified by the four methods. One-sided Mann-Whitney U test is used to calculate the p-values. Violin plots show quartiles and full data distribution (violin shape). **g** Venn plot comparing the SVGs from four methods and the DEGs selected based on cortical layer annotations by DEseq2. SVGs identified by SEPAR show notably greater overlap with DEGs than those detected by STAMarker, SPARK-X, or SpatialDE. **h** An illustration showcasing the effects of SEPAR refinement on 6 marker genes. Expression values are normalized to [0,1] within each panel for visualization. **i** Violin plots comparing the distribution of Moran’s I values for genes before and after refinement, with sample size being 3000 per condition. Violin plots show median (center line), interquartile range (box), and full data distribution (violin shape).

The dorsolateral prefrontal cortex (DLPFC) dataset^1^ was generated using the 10 × Visium platform. It consists of a total of 12 slices with manual annotations of the DLPFC layers and white matter (WM) (Fig. 2a). Each slice contains over 3000 spots, with more than 20000 genes measured. This dataset has been widely utilized for evaluating spatial domain identification techniques, and encompasses many biologically important SVGs linked to the structural layers of the cortex.

SEPAR effectively identifies key spatial metagene expression patterns that demonstrate strong biological coherence through cell-type and functional enrichment. Figure 2b highlights the 12 key patterns selected according to the pattern significance score (PSS), along with the number of pattern-specific genes. The smallest number of associated genes is 7 and the largest is 1210. We examined how each pattern maps to the manually annotated cortex layers (Supplementary Fig. 1). The violin plots illustrate the distribution of 12 spatial metagene expression patterns across manually annotated cortical layers (L1–L6) and white matter (WM), revealing the relationship between computationally extracted expression patterns and anatomically defined laminar organization. Patterns 1–8 exhibit a strong correspondence with the structural cortical layers. For instance, Pattern 2 correlates with layers L1 and WM, while Pattern 3 corresponds predominantly with WM. Notably, Patterns 10–12 reveal spatial organizations that do not conform to the classical laminar structure of DLPFC. Subsequent cell-type enrichment and gene ontology analyses confirm the biological relevance of these non-canonical patterns (Fig. 2c–d, Supplementary Figs. 2–3), which reveal their roles in extracellular organization, lipid metabolism, and membrane specialization, respectively. This demonstrates SEPAR’s ability in detecting meaningful metagene patterns beyond conventional layer-based analysis.

Cell-type enrichment analysis of the metagene expression pattern-specific genes shows distinct cellular compositions across spatial domains. Using CellGO^44^ with three established cell-type identifiers^45^, we examined the cellular preferences of each pattern. Analysis with major brain cell populations (ExN, InN, Oligo, OPC, Astro, and Microglia) (Fig. 2c) and detailed excitatory neuron (ExN) subtypes (L2/3IT, L3/5IT, L5/6NP, L6CT, L6IT, and L6b) in the prefrontal cortex (Fig. 2d) reveals cell-type-specific enrichment results. Patterns 1 and 4 are significantly associated with excitatory neurons, with *p*-values of 0 and 1.71e-128, respectively. Further analysis reveals layer-specific preferences, where Pattern 1 shows stronger enrichment for L3/5IT cells (*p*-value = 0), while Pattern 4 exhibits preferential enrichment for L2/3IT cells (*p*-value = 0), consistent with the spatial distribution of these patterns. Pattern 3 shows strong enrichment for oligodendrocytes (*p*-value = 0) and significant association with L6IT excitatory neurons (*p*-value = 2.23e-17), suggesting important neuron-glia interactions specific to deeper cortical layers. Additional analysis of inhibitory neuron subtypes (LAMP5, PVALB, SST, VIP, and SNCG) is presented in Supplementary Fig. 2.

Gene-ontology enrichment analysis of pattern-specific genes reveals distinct functional characteristics of each identified pattern Using g:Profiler^46^ (Supplementary Fig. 3). Pattern 10 shows significant enrichment in terms related to extracellular space (*p*-value = 1.11e-07) and anatomical structure morphogenesis (*p*-value = 9.06e-05). This gene set and the corresponding structure likely involve components of the extracellular matrix, growth factors, and signaling molecules, all of which are ubiquitously distributed and essential for maintaining the structure and function of the cortex. Pattern 11 reveals significant enrichment in lipid metabolic processes (*p*-value = 2.10e-10). Additional enrichment in regulation of lipid metabolic processes (*p*-value = 5.74e-09), lipid storage (*p*-value = 2.52e-07), and lipid catabolic processes (*p*-value = 2.28e-07) further underscores the critical role of lipid metabolism in this brain region. These findings reveal a distinct region predominantly in layer L1 of DLPFC with enhanced lipid catabolic activity, suggesting the presence of specialized cellular populations with elevated lipid metabolic processes, which may contribute to local neuronal function and energy homeostasis^47^. We note that Patterns 10 and 11 have not been identified by other existing methods.

SEPAR can identify the SVGs with high accuracy. We compared with the updated SVG identification methods: STAMarker^34^, SPARK-X^33^, and SpatialDE^10^. The UpSet plot in Fig. 2e visualizes that 359 SVGs were consistently identified across all four methods, which underscores substantial concordance. Notably, the greatest three-way overlap is between SEPAR, SpatialDE, and SPARK-X, with 435 common SVGs, indicating substantial agreement for specific SVG types. To further evaluate the performance of these methods, we calculated the spatial autocorrelation of uniquely identified SVGs using Moran’s I^48^. As shown in Fig. 2f, SVGs identified by SEPAR and SpatialDE exhibit significantly higher Moran’s I values (*p*-value = 1.42e-98 and *p*-value = 1.51e-06, respectively; one-sided Mann-Whitney U test) compared to background genes, with SVGs identified by SEPAR showing significantly higher Moran’s I values than those obtained using SpatialDE (*p*-value 1.75e-29). The SVGs uniquely identified by SEPAR have greater Moran’s I values than the median of the background. In contrast, SVGs identified by STAMarker and SPARK-X do not show statistically significant differences from the background genes. Here, background genes were defined as all genes excluding common SVGs identified by the four methods. We focused on genes expressed in 150–3,000 spots to optimally capture structured spatial patterns while avoiding sparse or ubiquitous expression profiles (Supplementary Fig. 4). This comparative advantage against all other methods underscores SEPAR’s enhanced sensitivity in detecting biologically relevant spatial patterns. Thereafter, we compared the SVGs with the 2903 differentially expressed genes (DEGs) computed based on cortical layer annotations (Layer 1–6 and white matter) using DESeq2^49^ (Fig. 2g). Among the 1,831 SVGs identified by SEPAR, 1,674 are DEGs, substantially surpassing the DEG counts identified by STAMarker (653), SPARK-X (1,337), and SpatialDE (964). Additionally, we compared SEPAR with NSFH^24^, a hybrid nonnegative spatial factorization method. The SVGs identified with NSFH contain only 194 DEGs (10.8%) (Supplementary Fig. 5). These results demonstrate SEPAR’s superior sensitivity and accuracy in identifying biologically meaningful SVGs. We further compared the identified SVGs with 66 canonical layer-specific marker genes obtained from previous studies^1,50,51^. Notably, 27 genes overlap with the identified SVGs. The overlapping genes exhibit significantly higher Moran’s I values compared to the remaining 39 non-overlapping markers (*p*-value < 0.01, one-sided Mann-Whitney U test) as shown in Supplementary Fig. 6, which highlights the distinct aims of marker gene selection and SVG detection. Further, we also compared the SVGs with the highly variable DEGs (defined as the intersection of the DEGs set and the top 3000 HVGs identified using Scanpy package^52^ from the original data) (Supplementary Fig. 7). The SVGs detected by SEPAR show the largest intersection (891 genes), corroborating that SEPAR’s unsupervised SVG detection retains a considerable amount of spatial information. To validate the practical utility of identified SVGs, we employed the 1831 SVGs as input for the spatial clustering methods STAGATE^14^ and BayesSpace^13^, and compared with their default setting (STAGATE: 3000HVGs, BayesSpace 2000HVGs). As depicted in Supplementary Fig. 8, utilizing the selected SVGs enhances clustering performance. The ARI (Adjusted Rand Index^53^, a clustering accuracy metric that adjusts for chance agreement between partitions), increases from 0.52 to 0.54 for STAGATE and from 0.46 to 0.52 for BayesSpace. This improvement further affirms the efficacy of the SVGs identified by SEPAR.

SEPAR also demonstrates robust performance in two key downstream applications: gene expression refinement and spatial domain identification. For gene refinement, we selected six marker genes: *CUX2* (a marker for upper layer neurons), *GFAP* (expressed in astracytes), *SLC17A7* (expressed in excitatory neurons), *RORB* (expressed in layer 4), *PLP1* (a key marker for oligodendrocytes), and *FOXP2* (specifically expressed in deep cortical layers neurons)^54,55^. Figure 2h illustrates the original expression and that refined with SEPAR for these genes, where SEPAR effectively reduces noise, thereby enhancing the accurate representation of underlying biological signals. Figure 2i reveals the differences in Moran’s I distribution for all genes before and after refinement, demonstrating a substantial overall increase in Moran’s I.

For spatial clustering, we summarized the results of SEPAR, STAGATE^14^, GraphST^15^, and BASS^16^ on 12 DLPFC samples (Supplementary Fig. 9 and Supplementary Fig. 10). Additionally, direct comparison of spatial clustering using NMF-based methods demonstrates SEPAR’s superior performance with ARI = 0.553 and Moran’s I = 0.884, substantially outperforming NSFH (ARI = 0.364, Moran’s I = 0.856) and SpiceMix (ARI = 0.163, Moran’s I = 0.409) (Supplementary Fig. 11). SEPAR effectively identifies the layer structure in the samples, and performs comparably to algorithms specially designed for clustering.

Collectively, applying SEPAR to the DLPFC dataset confirms SEPAR’s robust performance in spatial metagene expression pattern identification, pattern-specific gene analysis, SVG detection, gene expression denoising and spatial domain identification.

### SEPAR identifies distinct laminar structures and spatial patterns in mouse olfactory bulb

**Fig. 3:**
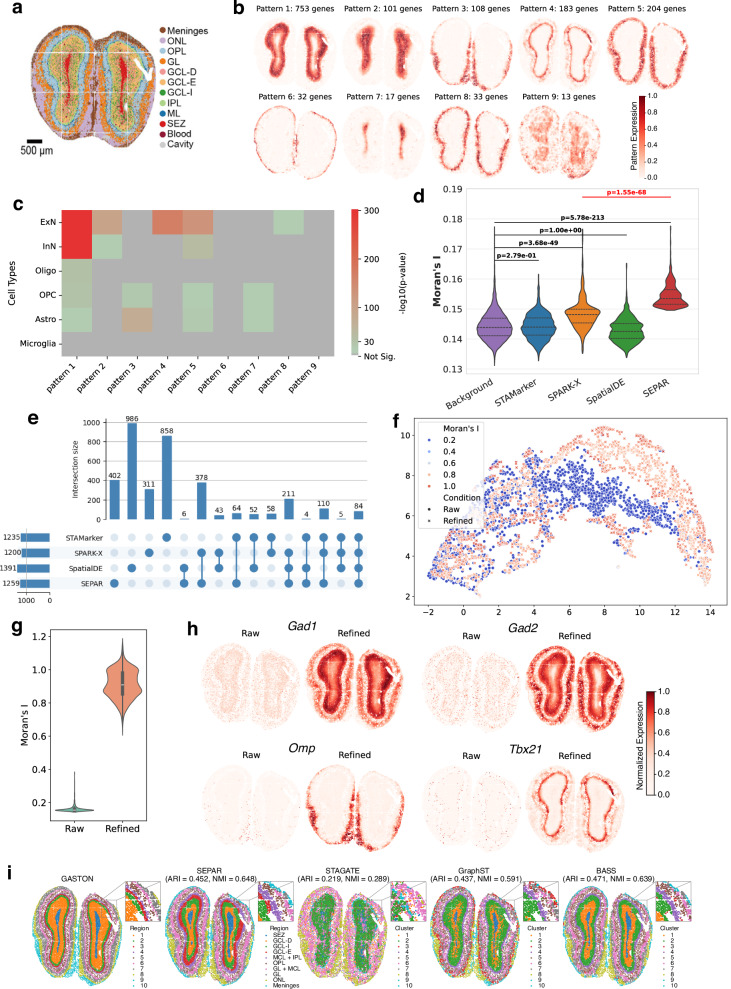
Analysis of the mouse olfactory bulb SRT data^40^ generated by Stereo-seq. **a** Unsupervised spatial clustering results adapted from Chen et al.^40^ under CC-BY license. **b** Nine identified spatial patterns of metagenes with the corresponding pattern-specific genes. **c** Cell-type enrichment analysis on the gene sets corresponding to each pattern using CellGO. **d** Comparison of Moran’s I values for uniquely identified SVGs by each method. The sample sizes of background, STAMarker, SPARK-X, SpatialDE and SEPAR are 10,527; 744; 302; 442; and 396, respectively. Background genes are all genes excluding the common SVGs identified by the four methods. One-sided Mann-Whitney U test is used to calculate the p-values. Violin plots show quartiles and full data distribution (violin shape). **e** UpSet plot for SVGs identified by STAMarker, SPARK-X, SpatialDE and SEPAR. **f** UMAP plot of gene expression before and after refinement, colored by Moran’s I and shaped by condition. **g** Violin plot for Moran’s I of genes before and after gene expression refinement with sample size being 2000 per condition. Violin plots show median (center line), interquartile range (box), and full data distribution (violin shape). **h** Visualization of 4 marker genes before and after gene expression refinement. Expression values are normalized to [0,1] within each panel for visualization. **i** Comparison of spatial domains identified by SEPAR, STAGATE, GraphST, and BASS against GASTON reference labels.

We applied SEPAR to analyze the spatial organization of the mouse olfactory bulb using Stereo-seq SRT data^40^. The olfactory bulb exhibits a well-defined laminar architecture, comprising multiple distinct layers from the center outward: the subependymal zone (SEZ), granule cell layer (GCL), internal plexiform layer (IPL), mitral cell layer (MCL), outer plexiform layer (OPL), glomerular layer (GL), olfactory nerve layer (ONL), and meninges. These anatomical layers were previously identified in the original study through spatially constrained clustering (SCC), which integrated both spatial proximity and transcriptomic similarity in a graph-based approach (Fig. 3a)^40^. Our analysis using SEPAR successfully recapitulated these known anatomical structures while providing additional insights into their molecular characteristics.

SEPAR identifies 30 spatial metagene expression patterns with the 9 highest-scoring patterns in Fig. 3b. The number of pattern-specific genes ranges from 13 to 753, and these patterns reveal predominantly distinct laminar structures, with Patterns 5 and 8 converging on the granular layer while capturing different molecular signatures within this anatomical region. We examined how each pattern maps to the annotated layers (Supplementary Fig. 12). We then performed cell-type enrichment analysis using CellGO and GO term enrichment using g:Profiler for the pattern-specific gene sets (Fig. 3c and Supplementary Fig. 13). The gene set for Pattern 7 is closely linked to processes such as “regulation of neuron differentiation" (GO:0045664, *p*-value = 2.97e-04) and “neurogenesis" (GO:0022008, *p*-value = 4.53e-04), both of which are critical to the SEZ, a recognized neurogenic niche. The enrichment of “oligodendrocyte differentiation" (GO:0048709, *p*-value = 1.21e-03) supports the cell-type enrichment results that astrocytes and oligodendrocyte precursor cells are abundant in this region, consistent with its role in ongoing neural development and repair. Pattern 6, meanwhile, is characterized by terms like “collagen-containing extracellular matrix" (GO:0062023, *p*-value = 3.76e-11) and “extracellular matrix structural constituent conferring compression resistance" (GO:0030021, *p*-value = 8.75e-05). The spatial location of Pattern 6 aligns with the meninges as identified in prior research^40^, demonstrating that SEPAR is a reliable tool for unsupervised identification of biologically important regions based on spatial distribution of decomposed patterns and subsequent gene-specific analysis.

According to the metagene patterns, SEPAR identifies 1259 SVGs (Fig. 3e). Compared with STAMarker, SPARK-X, and SpatialDE, 84 genes are identified in common, with SEPAR and SPARK-X sharing the largest overlap (378 genes), and SEPAR and STAMarker sharing the second largest overlap (64 genes). To evaluate the spatial variability of SVGs uniquely identified by each individual method, the Moran’s I values were compared (Fig. 3d). Compared to the background genes, the SVGs uniquely identified by SEPAR and SPARK-X exhibit significantly higher Moran’s I values (*p*-value = 5.78e-213 and *p*-value = 3.68e-29, respectively, one-sided Mann-Whitney U test), with SVGs identified by SEPAR showing significantly higher values than those using SPARK-X (*p*-value = 1.55e-68). The SVGs identified by SEPAR have higher Moran’s I values than the median of the background genes. In contrast, the SVGs identified using STAMarker and SpatialDE do not show statistically significant differences from the background genes, which were defined as all genes excluding the common SVGs identified by the four methods. Here genes expressed in 100–5000 spots were considered (Supplementary Fig. 14). Consistent with our previous findings, SEPAR demonstrates superior statistical significance across all compared methods, further confirming its enhanced capability to detect spatially structured gene expression patterns across different biological contexts.

Gene expression denoising using SEPAR demonstrates substantial improvement in spatial smoothness. Figure 3f presents the UMAP visualization of gene expression before and after refinement, showing minimal changes in distribution, indicating that the adjustments did not substantially distort the original data. Additionally, as shown in Fig. 3g, the spatial autocorrelation of gene expression increases substantially after refinement. We further examined several marker genes: *Gad1* and *Gad2* (Glutamate Decarboxylase 1 and 2) for GABAergic neurons, *Omp* (Olfactory Marker Protein) for mature olfactory sensory neurons, and *Tbx21* (*T-box 21*) for mitral and tufted cells^54,56^. The comparison of pre- and post-denoising in Fig. 3h demonstrates a marked enhancement in the spatial structure, particularly for *Tbx21*, whose post-denoising pattern aligns well with the mitral cell layer (MCL) of the olfactory bulb, predominantly composed of Mitral Cells.

Finally, we compared SEPAR’s clustering performance with STAGATE, GraphST, and BASS (Fig. 3i). For quantitative evaluation, we compared the identified spatial domains against those from GASTON^57^, an interpretable deep learning method that learns tissue topography and identifies spatial domains through isodepth modeling. SEPAR achieves superior performance in capturing tissue organization patterns with ARI = 0.452 and NMI = 0.648, where Normalized Mutual Information (NMI)^58^ is a measure of similarity between two clustering results that accounts for cluster size imbalances. It substantially outperforms STAGATE (ARI = 0.219, NMI = 0.289) and GraphST (ARI = 0.437, NMI = 0.591), while comparable to BASS (ARI = 0.471, NMI = 0.639). Visual inspection further confirms SEPAR’s ability to preserve sharp layer boundaries and distinctive olfactory bulb architecture, particularly evident in the detailed comparisons shown in Fig. 3i.

### SEPAR enables efficient spatial clustering and expression refinement for image-based spatial transcriptomics

Image-based spatial transcriptomics technologies that utilize fluorescence in situ hybridization or in situ sequencing, such as seqFISH+^59^, osmFISH^41^, MERFISH^5^, and STARmap^60^, can offer single-cell resolution expression and precise spatial information of RNA transcripts. These methods utilize pre-designed probes and high-resolution microscopy for detection, thus enabling absolute quantification of transcripts with spatial context, but they are restricted to limited pre-selected genes compared to sequencing-based spatial transcriptomics technologies. Given these characteristics, we emphasize spatial domain identification and gene expression refinement when using SEPAR for downstream analysis. We applied SEPAR to mouse somatosensory cortex dataset generated by osmFISH^41^ and mouse hypothalamic preoptic region dataset generated by MERFISH^5^.

**Fig. 4:**
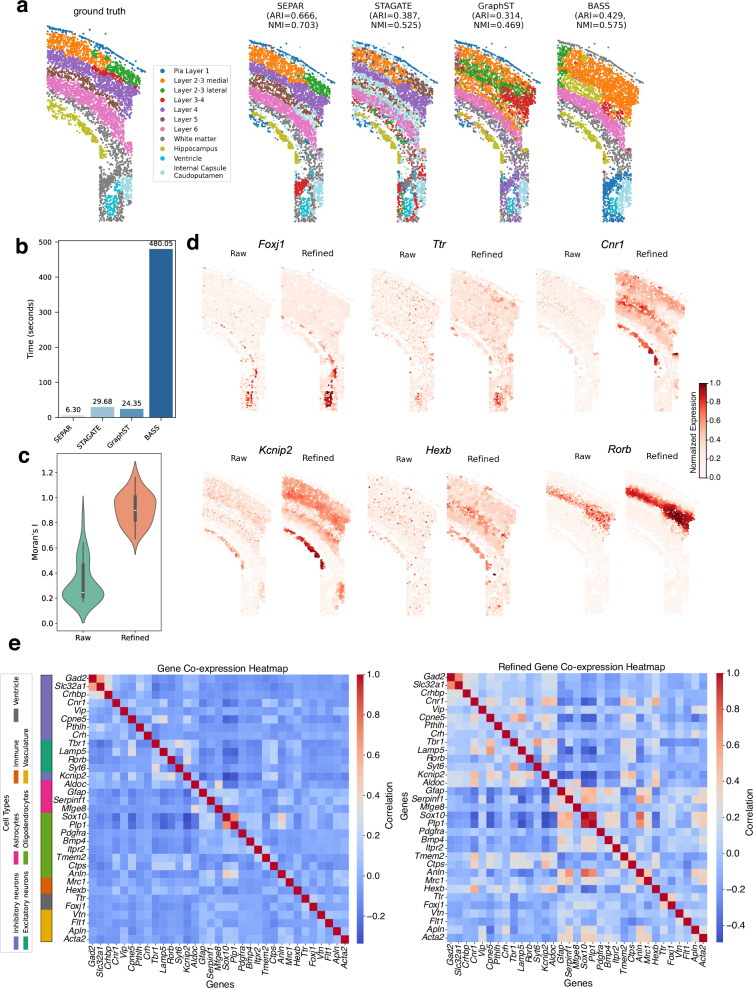
Analysis of the mouse somatosensory cortex data generated by osmFISH. **a** Spatial domain identification using SEPAR, STAGATE, GraphST and BASS. **b** Comparison of running time for SEPAR, STAGATE, GraphST and BASS. **c** Violin plots of Moran’s I values for gene expression before and after refinement with sample size being 33 per condition. **d** Visualization of 6 genes before and after gene expression refinement. Expression values are normalized to [0,1] within each panel for visualization. **e** Gene co-expression heatmaps generated from raw and refined expression data, with cell-type markers annotated based on original osmFISH study^41^. Violin plots show median (center line), interquartile range (box), and full data distribution (violin shape).

We first analyzed the osmFISH dataset from the mouse somatosensory cortex^41^, comprising 4,839 cells and 33 genes with  ~ 2 *μ* m spatial resolution. All 30 spatial patterns identified by SEPAR are shown in Supplementary Fig. 15. Figure 4a shows the spatial domains identified by different methods. SEPAR achieves superior results, with ARI = 0.666 and NMI = 0.703, greatly outperforming STAGATE (ARI = 0.387, NMI = 0.525), GraphST (ARI = 0.314, NMI = 0.469) and BASS (ARI = 0.429, NMI = 0.575). Since SEPAR is developed based on matrix factorization, the model training process is much faster than the neural network-based methods. As illustrated in Fig. 4b, SEPAR exhibits the shortest runtime among the four methods evaluated, requiring only 6.3 seconds – about one-quarter of the runtime of the second-fastest method GraphST.

Next, we performed gene refinement and spatial gene co-expression analysis, demonstrating that gene refinement helps detect more correlated genes. As observed in Fig. 4d, gene refinement notably reduces noise, enhancing cortical structure clarity and substantially improving Moran’s I values of the genes (Fig. 4c). After gene refinement, more spatially correlated genes are identified (Fig. 4e): 23 pairs show strong correlations (correlation coefficient  > 0.4) after refinement, whereas only 3 pairs have been observed in the raw data. As an example, genes *Foxj1* and *Ttr* show much higher correlations after gene refinement (Fig. 4d). According to the existing research^41^, both *Foxj1* and *Ttr* are marker genes for ependymal and choroid plexus cells, respectively. These cells are prominently distributed in the ventricle, showing anatomical adjacency and functional interactions. As another example, *Cnr1*, *Kcnip2* and *Hexb* show much clearer correlated patterns after applying SEPAR (Fig. 4d,e). Both *Cnr1* and *Kcnip2* relate to inhibitory neurons, while *Hexb* is associated with microglial immune cells. While the spatial pattern of *Hexb* is not visually apparent in Fig. 4d, violin plots of raw *Hexb* expression across spatial domains (Supplementary Fig. 16) confirm its elevated expression in the hippocampus and Layer 5, suggesting its role in neural plasticity and immune function. Despite having only 33 genes in the osmFISH dataset, SEPAR demonstrates its capability for effective analysis.

To further validate SEPAR’s performance on image-based technologies with larger gene panels, we analyzed a MERFISH dataset^42^ from mouse hypothalamic preoptic region measuring 155 genes across 5926 cells (Supplementary Figs. 17–21). SEPAR also achieves competitive spatial domain identification with superior computational efficiency, and the refined expression patterns show enhanced spatial coherence with improved gene co-expression discovery (Supplementary Note 2).

### SEPAR identifies the associations between genes and proteins in spatial CITE-seq data

Recent technological advances have enabled simultaneous profiling of multiple molecular modalities such as RNA, protein, and chromatin accessibility within the same spatial context, offering unprecedented opportunities to study the complex interplay between different molecular layers^11,12^. We next explored the capability of SEPAR in vertical integration of multi-omics data with two datasets generated by spatial CITE-seq and MISAR-seq, respectively.

**Fig. 5:**
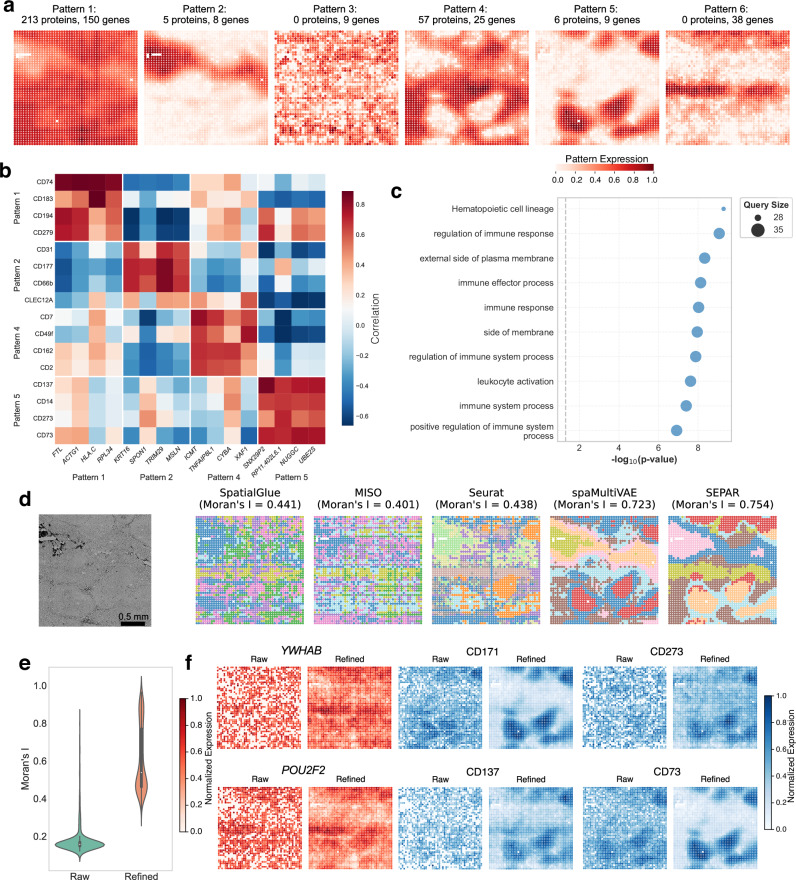
Integrative analysis of spatial CITE-seq data from human tonsil tissue using SEPAR. **a** Six spatial patterns identified by SEPAR within the spatial CITE-seq dataset, detailing the associated proteins and gene counts for each pattern. **b** Co-expression heatmap with denoised expression of selected genes and proteins from Pattern 1, 2, 4, and 5. **c** Enrichment analysis for Pattern 4 showing significant biological processes and pathways. **d** Comparison of spatial clustering. High-resolution microscope image (leftmost) provides reference for tissue architecture, followed by clustering results from SpatialGlue, MISO, Seurat, spaMultiVAE, and SEPAR, with corresponding Moran’s I values indicating spatial autocorrelation performance. **e** Violin plots of Moran’s I value for genes and proteins before and after refinement with sample size being 1,267 per condition. **f** Visualization of 2 genes (red) and 4 proteins (blue) before and after refinement. Expression values are normalized to [0,1] within each panel for visualization. Violin plots show median (center line), interquartile range (box), and full data distribution (violin shape).

First, SEPAR was applied to a spatial CITE-seq dataset from human tonsil tissue^11^. The dataset contains 2492 spots with 25 *μ* m resolution, measuring 984 spatially informative genes (selected from a total of 28,417 genes) and 283 proteins. Spatial CITE-seq enables simultaneous measurement of gene expression and protein levels, providing complementary molecular information. Here, the metagenes are extended to meta-gene-proteins. Six spatial patterns corresponding to meta-gene-proteins identified are displayed in Fig. 5a. Among these, four patterns: Pattern 1, 2, 4, and 5 incorporate cross-omics information.

We hypothesized that these spatially matched gene-protein sets harbor potential gene-protein relationships. To validate this hypothesis, we selected the four top-ranking genes and proteins with highest PSS score from Pattern 1, 2, 4, and 5, and generated a co-expression heatmap with denoised expression level (Fig. 5b). The analysis reveals higher correlation coefficients between genes and proteins within the same pattern (mean  = 0.622) compared to those between different patterns (mean = − 0.086). Despite the inherent heterogeneity between protein and gene data, SEPAR successfully extracts shared patterns, linking RNA with protein data. For example, the co-expression between CD74 and *HLA-C* identified in our analysis is consistent with established biological mechanisms, where CD74 has been shown to regulate both MHC class II and certain MHC class I molecules in antigen presentation^61^. To further examine the spatial coordination between genes and proteins within pattern groups, we visualized representative molecular features from each pattern, which show generally consistent spatial distributions within pattern groups (Supplementary Fig. 22). Furthermore, enrichment analysis was performed on the identified pattern-specific genes and proteins, with results for Pattern 4 shown in Fig. 5c. The enrichment analysis reveals significant immune-related functions, particularly in membrane-associated components and immune response pathways. Notably, the enrichment of “external side of plasma membrane" and “cell surface" components, along with immune response pathways and hematopoietic cell lineage, indicates robust immune cell activities and extensive cell-cell interactions in the tissue microenvironment.

The spatial patterns corresponding to meta-gene-proteins identified using SEPAR also enable unsupervised spatial clustering. We benchmarked SEPAR against established spatial multi-omics integration methods including SpatialGlue^35^, MISO^36^, Seurat^62^, and spaMultiVAE^18^ (Fig. 5d). SEPAR achieves the highest spatial autocorrelation (Moran’s I = 0.754), followed by spaMultiVAE (0.723), while SpatialGlue (0.441), MISO (0.401), and Seurat (0.438) show lower spatial coherence. The clustering results reveal that SEPAR and spaMultiVAE both capture contiguous spatial domains, whereas SpatialGlue, MISO, and Seurat produce more fragmented patterns with reduced spatial continuity. This demonstrates SEPAR’s effective integration of cross-omics information for biologically meaningful spatial domain identification. To evaluate the contribution of individual modalities, we compared SEPAR with GraphST on RNA-only, protein-only, and a combination of both (using 7 clusters consistently for fair comparison), with SEPAR consistently outperforming GraphST across all conditions (RNA-only: 0.629 vs 0.492; protein-only: 0.782 vs 0.629; integrated RNA-protein: 0.754 vs 0.466) (Supplementary Fig. 23). In this dataset, protein expression exhibits stronger spatial structure than RNA, as reflected in the higher protein-only Moran’s I value, consistent with CITE-seq’s technical characteristics where proteins show lower dropout rates ( ~ 12%) than RNA ( > 80%)^63^. While SEPAR’s integrated analysis shows slightly lower spatial autocorrelation than protein-only, it produces more balanced domain boundaries that better reflect the complementary information from both modalities, with the combined Moran’s I (0.754) falling between the two individual modalities, suggesting biological value in multi-omics integration beyond simple spatial coherence metrics.

As for the refinement effect, quantitative assessment using Moran’s I statistic demonstrates substantial enhancement in spatial coherence for both gene and protein expressions (Fig. 5e). The refinement process substantially improves data quality (Fig. 5f), where the initially noisy and ambiguous gene expression patterns are transformed into well-defined spatial distributions with intensified characteristic features. This enhanced data quality enables comprehensive spatial co-expression analysis of the top 15 highly variable genes and proteins (Supplementary Fig. 24), revealing markedly improved molecular correlations: 44 pairs of biological molecules showed strong correlations (correlation coefficient  > 0.5) after refinement, whereas no such strong correlations have been observed in the raw data. For example, the co-expression patterns between genes *YWHAB* and *POU2F2*, and the proteins CD71, CD137, CD73, and CD273 (Fig. 5f), demonstrate SEPAR’s ability to detect cross-modality relationships after refinement. Comparison with spaVAE^18^ further validates these refinement results, showing reduced artifacts and better preservation of spatial structures (Supplementary Fig. 25).

### SEPAR identifies the associations between gene expression and ATAC in MISAR-seq data

**Fig. 6:**
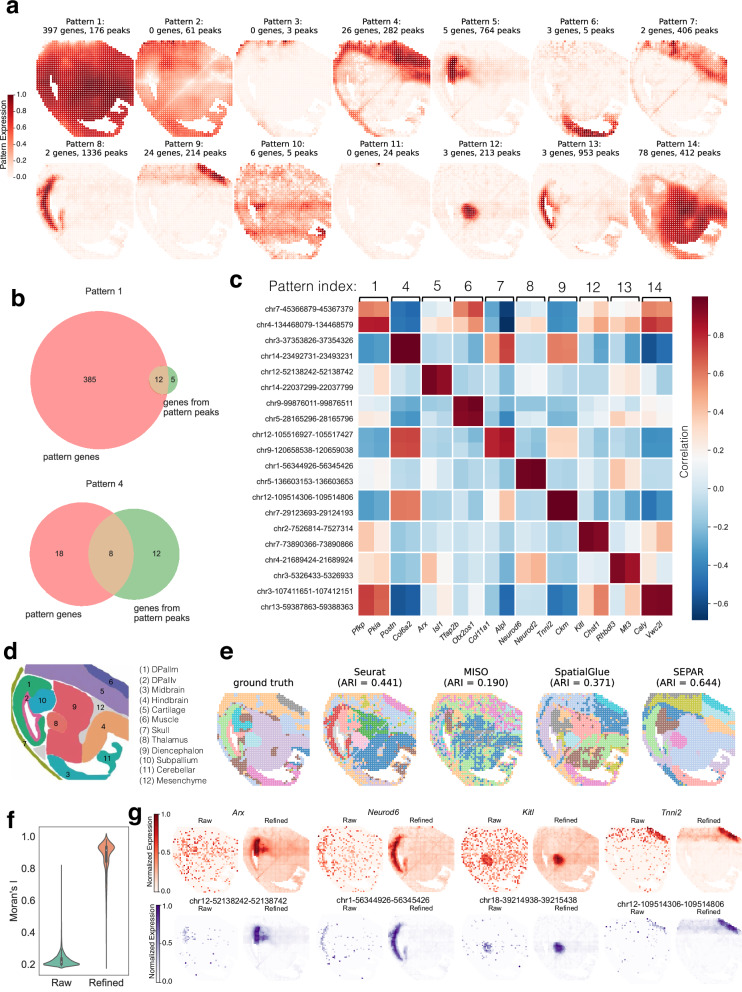
Analysis of MISAR-seq data from mouse embryonic (E15.5) brain tissue using SEPAR. **a** Fourteen spatial patterns identified by SEPAR within the MISAR-seq dataset, detailing the associated peaks and gene counts for each pattern. **b** Gene-peak association analysis showing overlap between pattern-specific gene sets and pattern-specific peaks' associated genes. **c** Co-expression heatmap with denoised expression of selected genes and peaks from identified spatial patterns. **d** Reference annotation map of mouse embryonic brain regions. **e** Clustering comparison against ground truth derived from the original MISAR-seq analysis. Left to right: ground truth, clustering results from Seurat, MISO, SpatialGlue, and SEPAR. **f** Violin plots of Moran’s I values for gene and peak expression before and after refinement with sample size being 5000 per condition. **g** Visualization of four genes (red) and four peaks (purple) before and after refinement. Expression values are normalized to [0,1] within each panel for visualization. Violin plots show median (center line), interquartile range (box), and full data distribution (violin shape).

We further applied SEPAR directly for integrating spatial ATAC-seq and SRT data on a mouse embryonic (E15.5) brain dataset obtained through MISAR-seq^12^. MISAR-seq is a technology that simultaneously profiles chromatin accessibility and gene expression within the spatial context at 50 *μ* m resolution, providing insights into gene regulation mechanisms. The dataset contains 1,949 spots, and after selecting spatially informative features, our analysis included 4,460 ATAC peaks and 540 genes. The 14 key spatial patterns corresponding to meta-gene-peak identified are displayed in Fig. 6a, with 11 patterns linked to both ATAC and RNA data, indicating SEPAR’s capability in effectively connecting RNA with chromatin accessibility. To validate these connections, the genes associated with the pattern-specific peaks were first identified using ChIPseeker^64^ with those absent from the gene expression data deleted. This gene set was then compared with the previously identified pattern-specific genes. The comparison reveals meaningful overlaps: in Pattern 1, 12 out of 17 genes identified from the pattern-specific peaks are in the pattern-specific gene set, while in Pattern 4, 8 genes overlap between 26 pattern-specific genes and 20 genes obtained from pattern-specific peaks (Fig. 6b), suggesting spatially coherent patterns between chromatin accessibility and gene expression, revealing spatial correlations between co-localized molecules that provide computational evidence warranting further experimental investigation. Spatial visualization of representative patterns further demonstrates consistent co-localization between pattern-specific genes and peaks across multiple patterns (Supplementary Fig. 26). We selected two top-ranking pattern-specific genes and peaks from each of the 10 patterns and generated a co-expression heatmap with denoised expression level (Fig. 6c). The analysis reveals higher correlations between genes and peaks within the same pattern (mean = 0.613) compared to those between different patterns (mean = 0.028), demonstrating the expression-level concordance of genes and peaks identified using SEPAR.

We benchmarked SEPAR against established spatial multi-omics integration methods including Seurat, MISO, and SpatialGlue on spatial domain identification. Figure 6d shows the anatomic annotation of major tissue regions based on H&E images, while Fig. 6e compares the clustering results against ground truth derived from the original MISAR-seq analysis. SEPAR achieves the highest agreement with ground truth annotations (ARI = 0.644), outperforming SpatialGlue (0.371), Seurat (0.441), and MISO (0.190). SEPAR accurately identifies key brain regions including midbrain and thalamus, as well as surrounding non-neural tissues like muscle. Notably, SEPAR’s multi-omics integration produces more coherent spatial domains with clearer structural boundaries compared to competing methods, demonstrating the biological relevance of integrating RNA and ATAC-seq data for comprehensive tissue architecture characterization. Additional comparison with GraphST across individual and multiple modalities (using 12 clusters consistently for fair comparison) shows SEPAR’s consistent advantages in spatial autocorrelation (Integrated: Moran’s I = 0.870 vs 0.799; ATAC-only: 0.816 vs 0.732) and effective multi-omics integration, with integrated analysis (ARI = 0.644) outperforming individual modalities (RNA-only: ARI = 0.415; ATAC-only: ARI = 0.411) (Supplementary Fig. 27).

The refinement process leads to notably improved spatial autocorrelation as demonstrated by Moran’s I statistic (Fig. 6f). Spatial co-expression analysis of the top 15 highly variable genes and peaks (Supplementary Fig. 28) reveals improved molecular relationships: 137 pairs shows strong correlations (correlation coefficient  > 0.5) after refinement, whereas no such correlations have been observed in the raw data. Several notable co-expression pairs are identified, for example, genes *Col1a2*, *lgf2*, *Airn* with their corresponding peaks chr10-80260918-80261418, ch8-123411167-123411667, and chr7-141949425-141949925 (Fig. 6g). These multi-omics pairs, which share similar spatial patterns and are previously masked by noise in the raw data, become apparent after refinement. Additionally, analysis of spatially variable genes and peaks (Supplementary Figs. 29,30) corroborates the identified cross-omics spatial patterns.

To further characterize the regulatory mechanisms underlying these spatial patterns, we performed motif enrichment analysis for all pattern-specific peaks using HOMER (Hypergeometric Optimization of Motif EnRichment)^65^ (Supplementary Fig. 31). This analysis reveals distinct transcription factor binding motifs enriched in different spatial domains. Pattern 8, localized in the developing pallium (DPallm), shows enrichment of proneural basic helix-loop-helix (bHLH) transcription factor binding sites, including NeuroD1, NeuroG2, and Atoh1. For this pattern, we identified only two pattern-specific genes: *Neurod2* and *Neurod6*, both of which are essential for proper neuronal migration and differentiation^66^. The spatial co-occurrence of NeuroD1 and NeuroG2 binding motifs and the expression of Neurod2 and Neurod6 in the developing pallium align with their known roles in neuronal differentiation^66,67^, though their regulatory relationships remain to be determined. Pattern 9, localized in the developing muscle tissue surrounding the E15.5 mouse brain, exhibits highly significant enrichment of E-box motifs (CANNTG) (Supplementary Fig. 31), which are canonical binding sites for myogenic regulatory factors (MRFs)^68^. The most significantly enriched motifs are from the bHLH family of myogenic regulatory factors, with Myf5 showing the strongest enrichment (*p*-value = 1e-66, 78.30% of target sequences), followed by MyoD (*p*-value = 1e-59, 78.77%) and MyoG (*p*-value = 1e-56, 85.85%). This pattern’s specific genes comprise a comprehensive set of muscle structural components, including myosin heavy chains (*Myh3*, *Myh8*), troponins (*Tnnt1*, *Tnnt3*, *Tnni1*, *Tnni2*, *Tnnc2*), and other essential sarcomeric proteins (*Acta1*, *Actc1*, *Des*, *Ttn*, *Mybpc1*, *Neb*). The extensive presence of MRF binding motifs in pattern-specific peaks, together with these muscle-specific genes, suggests active regulatory relationships in this muscle domain. Notably, TCF21 binding motifs show strong enrichment (*p*-value = 1e-59, 83.02% of target sequences) in pattern-specific peaks, along with TCF12 (*p*-value = 1e-52, 82.08%). TCF12 (also known as HEB) preferentially expresses in developing muscle and forms heterodimers with MyoD to regulate muscle-specific genes^69,70^, while TCF21 directly binds to regulatory regions of both Myf5 and MyoD to function as their upstream activator^71^. The presence of these functionally diverse but muscle-related genes, along with their experimentally validates regulatory interactions, demonstrates the potential of SEPAR’s multi-omics pattern-specific analysis in identifying comprehensive tissue-specific regulatory networks.

### Enhanced multislice integration with SEPARmult

Building upon the single-slice analysis, we extended SEPAR to simultaneously process multiple tissue sections called SEPARmult (“Methods”). This multislice approach leverages shared molecular patterns across adjacent sections while preserving slice-specific spatial relationships.

**Fig. 7:**
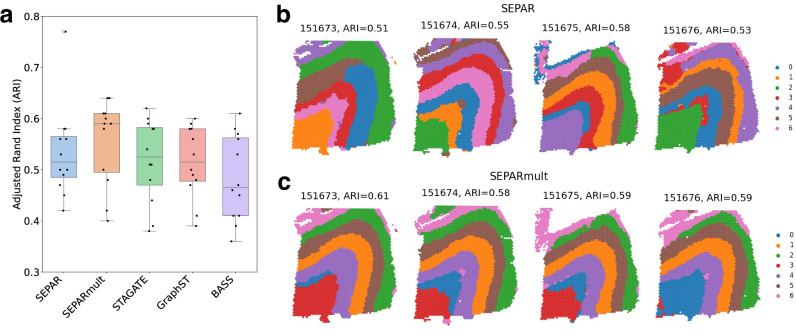
Performance evaluation of SEPARmult on DLPFC dataset. **a** Comparison of clustering performance across methods. Boxplots show ARI distributions for spatial domain identification using SEPAR, SEPARmult, STAGATE, GraphST, and BASS across 12 tissue sections. **b** Single-slice results for consecutive sections 151673--151676 using SEPAR. **c** Results for the same sections, showing improved consistency in domain boundaries using SEPARmult. Box plots show median (center line), first and third quartiles (box boundaries), and whiskers extending to 1.5 × interquartile range.

We applied SEPARmult to all 12 DLPFC sections and compared its performance against single-slice SEPAR and existing multislice methods. SEPARmult achieves a median ARI of 0.590, representing a considerable improvement over single-slice SEPAR (median ARI = 0.515) and outperforming STAGATE (0.525), GraphST (0.515), and BASS (0.465) across all sections (Fig. 7a). To visualize this improvement, we examined four consecutive sections (151673, 151674, 151675, 151676). SEPARmult produces more consistent spatial domain boundaries across sections (Fig. 7c) compared to individual single-slice analyses (Fig. 7b), demonstrating enhanced stability through cross-section information integration.

## Discussion

In this work, we introduced SEPAR, a robust method designed to identify spatial patterns of metagene expression along with associated pattern-specific gene sets for SRT data. SEPAR is a graph-regularized NMF-based approach that leverages the non-negative nature of gene expression data and NMF’s interpretability to uncover underlying patterns and structures for gene subsets in SRT data.

Using the identified spatial metagene expression patterns, SEPAR excels in various downstream analytical tasks, including spatial pattern-specific gene analysis, SVG identification, spatial clustering, and gene expression denoising. Unlike traditional methods for spatial domain identification that focus on expression patterns of all genes, SEPAR naturally groups genes into different patterns corresponding to metagenes. This holistic approach allows SEPAR to identify genes with better cell-type enrichment and gene ontology enrichment and strong regional differential expression without supervision.

A key advantage of SEPAR is its versatility across different data types. Beyond demonstrating robust performance in diverse spatial transcriptomics technologies (10 × Visium, Stereo-seq, osmFISH and MERFISH), where SEPAR consistently identified biologically meaningful spatial patterns and revealed tissue-specific expression programs across different resolution scales and measurement principles, SEPAR effectively handles multi-omics spatial molecular data. It identified spatially coherent patterns between RNA and protein expression in spatial CITE-seq data, as well as those between chromatin accessibility and gene expression in MISAR-seq data, revealing spatial correlations between co-localized molecules that provide computational evidence warranting further experimental investigations.

SEPAR demonstrates computational efficiency suitable for large-scale datasets. Analysis of a colorectal cancer VisiumHD dataset with over 500,000 spots completed within 1,000 seconds with linear scaling (Supplementary Note 3, Supplementary Fig. 32). Comparative analysis shows SEPAR is over 10 times faster than NSFH and over 100 times faster than SpiceMix. Beyond well-structured tissues, SEPAR effectively analyzes heterogeneous disease contexts, identifying 12 distinct spatial patterns capturing key cancer processes in the colorectal cancer dataset (Supplementary Note 1, Supplementary Figs. 33, 34).

While SEPAR’s expression refinement demonstrates robust performance, we validated against potential over-smoothing concerns through consistency analysis across datasets and cross-technology comparison (Supplementary Note 4, Supplementary Figs. 21, 25 and 35). SEPAR-refined Visium data showed enhanced concordance with high-resolution Xenium patterns, providing evidence for biological validity while acknowledging challenges for extremely sparse genes.

Several limitations warrant acknowledgment. Detection of very thin anatomical structures remains challenging due to spatial transcriptomics resolution limits and difficulty in aligning gene expression boundaries with morphological annotations-a challenge shared across spatial analysis methods, as recent benchmarking shows no method achieves ARI above 0.55 on manually annotated cortical layers^72^. For multi-omics data, spatial co-localization represents correlation rather than causation, requiring experimental follow-up for validation. We have extended SEPAR to SEPARmult for simultaneous multislice analysis. It works well for adjacent serial sections or technical replicates with minimal batch effects, but may be limited when biological or technical variations between samples violate the shared pattern assumption. While preprocessing with batch correction methods can mitigate this issue, it adds complexity. Future development of computational methods with integrated batch effect removal would enable broader application.

While SEPAR is applicable to various scenarios, several promising research directions emerge. First, incorporating single-cell RNA sequencing data and cell-type annotations could provide deeper biological insights, enabling simultaneous domain identification and cell-type deconvolution. Second, the flexible framework of SEPAR creates opportunities for incorporating biological prior knowledge such as gene regulatory networks to identify coordinated spatial patterns of transcription factors and their targets. Third, the principles underlying SEPARmult’s success could be extended to cross-platform integration of spatial transcriptomics data, addressing the critical challenge of different technical characteristics and batch effects across platforms.

## Methods

### Spatial metagene expression pattern recognition model

We developed a graph regularized non-negative matrix factorization (NMF)-based model to conduct Spatial metagene Expression PAttern Recognition (SEPAR). The model is formulated as:

1 $$\mathop{\min }\limits_{W\ge 0,H\ge 0}\parallel X-WH{\parallel }_{F}^{2}+\alpha {{\rm{tr}}}\,(\Omega {W}^{T}LW)+\beta \parallel W{\parallel }_{1}+\frac{\gamma }{2}\mathop{\sum }\limits_{i < j}{(\langle {h}_{i},{h}_{j}\rangle )}^{2}.$$

Here, *X* is the *n* × *p* gene expression matrix corresponding to *n* spots (cells) and *p* genes. *W* = {*w*_1_, *w*_2_, …, *w*_*r*_} represents the *r* spatial metagene expression patterns with *w*_*i*_ being the *i*-th column of *W*, and matrix *H* has size *r* × *p*, where each row vector *h*_*i*_ (for *i* = 1, …, *r*) indicates the *i*-th metagene expression signature across the *p* genes. *L* is the graph Laplacian matrix incorporating the spatial location and gene expression information. The weight matrix *Ω* is taken to be the diagonal matrix of Pattern Significance Scores (diag(*PSS*)), which measures the influence of the graph regularization for spatial metagene expression patterns. *α*, *β*, and *γ* are parameters controlling the weight of each regularization term. The weighted graph penalty term ensures that close spots are more likely to have similar pattern representations. The sparsity penalty in (1) helps identify localized smooth spatial patterns. And the final term promotes orthogonality between the metagenes *h*_*i*_ and *h*_*j*_ in matrix *H*, and minimizing this term reduces redundancy, guaranteeing pattern distinctness.

#### Graph construction

An undirected graph is built based on a predefined radius r^*^ or a computed radius:

2 $${r}^{* }=\lambda \sqrt{\frac{({x}_{max}-{x}_{min})({y}_{max}-{y}_{min})}{n}},$$

where *x*_*max*_, *x*_*min*_, *y*_*max*_, and *y*_*min*_ represent the maximum and minimum values of the 2D coordinates for all spots/cells, and *λ* is a scaling parameter, typically set to *λ* = 1.2. The adjacency matrix *A* of the graph is defined as:

$${A}_{ij}=\left\{\begin{array}{rlr}{s}_{ij} & \,\,{{\rm{if}}}\,\,{d}_{euc}(i,j) < {r}^{* }, & \\ 0 & \,\,{{\rm{else}}} \, . \hfill \end{array}\right.$$

Here, *d*_*euc*_(*i*, *j*) is the Euclidean distance between spatial locations of spots *i* and *j*. The weight *s*_*ij*_ for the edge between spots *i* and *j* is determined by the cosine distance *d*_*cos*_(*X*_*i*⋅_, *X*_*j*⋅_) between their gene expression profiles:

$$\begin{array}{r}{s}_{ij}=\exp (-\frac{{d}_{cos}^{2}({X}_{i\cdot },{X}_{j\cdot })}{2{d}^{* 2}}),\,{d}^{* }={Q}_{\frac{1}{{n}_{c}}}({d}_{cos}(:,:)),\end{array}$$

where ${Q}_{1/{n}_{c}}$ is the 1/*n*_*c*_ quantile, and *n*_*c*_ is the estimated number of clusters in the data. The Laplacian matrix *L* is then computed as *L* = *D* − *A*, where *D* is a diagonal matrix with the diagonal entries being the degree of the corresponding spot.

#### Pattern significance score (PSS)

To quantitatively evaluate the biological relevance and spatial coherence of extracted metagene expression patterns, we developed a metric PSS to measure their significance. For any given pattern *i*, we identified its corresponding spatial distribution *w*_*i*_ (the *i*-th column of the factorization matrix *W*) and the coefficient *h*_*i*_ (the *i*-th row of matrix *H*) for each gene. The pattern significance score (PSS) is defined as:

3 $$\begin{array}{rlr}{p}_{i} & =\frac{{X}^{T}{w}_{i}}{\parallel {w}_{i}{\parallel }_{1}},\,PS{S}_{i} & =\frac{\langle {p}_{i},{h}_{i}\rangle }{\parallel {p}_{i}{\parallel }_{2}\parallel {h}_{i}{\parallel }_{2}}.\end{array}$$

Here, *p*_*i*_ represents the average gene expression profile across all spatial locations where pattern *i* is active, and *PSS*_*i*_ measures the cosine similarity between this spatial expression profile and the pattern’s gene signature *h*_*i*_. A higher value of *PSS*_*i*_ indicates that the *i*-th gene expression pattern represented by *w*_*i*_ has strong correspondence between its gene signature and the actual expression observed in its active spatial locations, suggesting genuine biological relevance.

We incorporate PSS values into our spatial regularization framework to adaptively control smoothing intensity. In the following optimization process, patterns with lower PSS values receive weaker spatial regularization through smaller *PSS*_*j*_ coefficients, thereby protecting subtle biological patterns from over-smoothing while preventing noise patterns from being artificially smoothed into spurious spatial structures, allowing them to be eliminated through reconstruction error and sparsity constraints.

We iteratively solve the optimization problem (1). Following the multiplicative update framework, we derive the following update rules that guarantee non-negative solutions and monotonically decreasing of the objective function:

$$\begin{array}{rc} & {W}_{ij}\leftarrow {W}_{ij}\cdot \frac{{(X{H}^{T})}_{ij}+\alpha PS{S}_{j}{(AW)}_{ij}}{{(WH{H}^{T})}_{ij}+\alpha PS{S}_{j}{(DW)}_{ij}+\beta },\\ & {H}_{ij}\leftarrow {H}_{ij}\cdot \frac{{({W}^{T}X)}_{ij}}{{({W}^{T}WH)}_{ij}+\gamma {\sum }_{k\ne i}\langle {h}_{i},{h}_{k}\rangle {H}_{kj}}.\end{array}$$

The whole computation procedure is summarized in Supplementary Note 5.

#### Multi-omics analysis

For integrated analysis of multiple data modalities (RNA, ATAC-seq, protein) from the same spatial locations, after we implement preprocessing for all the modalities, the processed data matrices are concatenated to form an integrated matrix *X* = [*X*_*RNA*_, *X*_*ATAC*_, *X*_*protein*_]. Then, SEPAR is applied to this integrated matrix to identify shared spatial patterns across modalities.

### Multislice integration with SEPARmult

SEPARmult extends SEPAR for simultaneous analysis of *M* tissue sections (*X*_*s*_, *s* = 1, …, *M*) from the same sample source. The key innovation lies in maintaining slice-specific spatial pattern matrices *W*_*s*_ while sharing a common metagene expression matrix *H* across all sections:

$$\mathop{\min }\limits_{{W}_{s}\ge 0,H\ge 0} 	 \mathop{\sum }\limits_{s=1}^{M}(\parallel {X}_{s}-{W}_{s}H{\parallel }_{2}^{2}+\alpha {{\rm{tr}}}\,({{\rm{diag}}}\,(PSS){W}_{s}^{T}{L}_{s}{W}_{s})+\beta \parallel {W}_{s}{\parallel }_{1})\\ 	 +\frac{\gamma }{2} \mathop{\sum }\limits_{i < j}{(\langle {h}_{i},{h}_{j}\rangle )}^{2}.$$

Here, ${X}_{s}\in {{\mathbb{R}}}^{{n}_{s}\times p}$ represents the gene expression matrix for slice *s* with *n*_*s*_ spots, ${W}_{s}\in {{\mathbb{R}}}^{{n}_{s}\times r}$ captures slice-specific spatial patterns, and $H\in {{\mathbb{R}}}^{r\times p}$ represents shared metagene signatures. Each slice maintains its own spatial graph with Laplacian matrix *L*_*s*_ constructed from slice-specific coordinates.

The optimization alternates between updating slice-specific matrices {*W*_*s*_} and the shared matrix *H* using multiplicative update rules derived from the Karush-Kuhn-Tucker conditions. For each *W*_*s*_, the update follows:

$${W}_{s}\leftarrow {W}_{s}\odot \frac{{X}_{s}{H}^{T}+\alpha {{\rm{diag}}}\,(PSS){A}_{s}{W}_{s}}{{W}_{s}H{H}^{T}+\alpha {{\rm{diag}}}\,(PSS){D}_{s}{W}_{s}+\beta {{\bf{1}}}},$$

where *A*_*s*_ and *D*_*s*_ are the adjacency and degree matrices for slice *s*. The shared matrix *H* is updated by aggregating information across all slices, enabling the model to identify consistent metagene patterns while accommodating slice-specific spatial variations.

### Downstream tasks

#### Pattern-specific gene detection

To identify the genes specifically associated with each spatial metagene expression pattern, we implement a two-step normalization process on the matrix *H*:

4 $$\Lambda ={{\rm{diag}}}\,(\parallel {w}_{1}{\parallel }_{2},\ldots ,\parallel {w}_{r}{\parallel }_{2}),\,{H}^{{\prime} }=\Lambda H,\,P{r}_{ij}={H}_{ij}^{{\prime} }/\mathop{\sum }\limits_{k=1}^{r}| {H}_{kj}^{{\prime} }| ,$$

where *Pr* represents the normalized pattern contribution matrix to the genes. Each column of *Pr* quantifies the relative contribution of all patterns to a specific gene. A gene is classified as pattern-specific when its corresponding entry in *Pr* exceeds a threshold ϵ_*_, indicating strong association with a particular spatial metagene expression pattern.

#### Selection of SVGs

SVGs are different from the pattern-specific genes in that they can be expressed on more than one metagene expression pattern. The SVGs are identified by evaluating the contribution of each spatial pattern to the gene expression. For each gene *i*, we compute the reconstruction error from the combination of identified patterns:

5 $$er{r}_{i}=\frac{\parallel {X}_{\cdot i}-W{H}_{\cdot i}{\parallel }_{2}}{\parallel {X}_{\cdot i}{\parallel }_{2}}.$$

Genes with *err*_*i*_ < *ϵ* are classified as SVGs, indicating their expression exhibits strong spatial organization that aligns with the discovered tissue architecture.

#### Spatial domain identification

The decomposed basis matrix $W\in {{\mathbb{R}}}^{n\times r}$ from SEPAR can be taken as a lower-dimensional representation for the spots or cells. Spatial domain identification is performed with *W*. While SEPAR is robust to the choice of dimensionality parameter *r* (typically set to 30 in practice), not all identified patterns necessarily represent biologically meaningful spatial organizations. Indeed, SEPAR’s ability to detect spatial patterns beyond conventional anatomical structures (e.g., non-canonical patterns that deviate from classical laminar organization) necessitates a systematic filtering approach to distinguish genuine biological signals from technical artifacts. Therefore, spatial domain identification is performed with *W* through a sequential pattern filtering process. Initially, we filter out *N*_1_ patterns with the smallest *ℓ*_2_-norms, which typically represent low abundance patterns. We further exclude *N*_2_ patterns with the lowest PSS to eliminate the potentially noisy components. The resulting filtered low-dimensional matrix W^*^ is then subjected to *k*-means clustering to identify distinct spatial domains.

#### Gene expression denoising

The denoised expression data is directly derived from the reconstructed gene expression matrix *WH*. This reconstruction captures the essential low-dimensional structure of the data, effectively filtering out noise.

#### Enrichment analysis

Gene sets and peak sets corresponding to each identified spatial pattern were subjected to comprehensive functional enrichment analysis. GO term enrichment analysis was performed using g:Profiler^46^. Cell type enrichment analysis was performed using CellGO^44^, a computational tool that identifies cell type-specific active pathways by modeling signal propagation through Gene Ontology hierarchical structures. CellGO evaluates whether genes in each identified spatial pattern show significant enrichment in cell type-specific expression profiles from single-cell reference data, connecting spatial patterns to specific cell populations while considering hierarchical organization of biological functions. For multi-omics datasets containing chromatin accessibility data, motif enrichment analysis was performed using HOMER (Hypergeometric Optimization of Motif EnRichment)^65^ to identify transcription factor binding motifs enriched in pattern-specific peaks, providing insights into potential regulatory mechanisms underlying spatial gene expression patterns.

### Data preprocessing

For data preprocessing, we employed distinct protocols for different spatial transcriptomics platforms. For sequencing-based and multi-omics datasets, we performed log-transformation and library size normalization using the package Scanpy^52^. For multi-omics datasets, each modality was processed independently following this protocol. We selected the top genes with highest spatial autocorrelation (measured by Moran’s I statistic) for downstream analysis. The sequencing-based spatial transcriptomics data were processed by averaging each spot’s expression with its adjacent neighbors before input to SEPAR. For image-based spatial transcriptomics datasets, which typically contain fewer genes, the raw expression matrices were used directly as input.

### Parameter settings

SEPAR employs a principled parameter selection framework that distinguishes between user-controlled and theoretically-informed default parameters. The spatial regularization weight (*α*) is intentionally preserved as a user-selectable parameter, with recommended values of *α* = 0.3–1.0 for spatial domain analysis emphasizing tissue-level patterns and *α* = 0.1–0.3 for high-resolution analysis identifying small niches. The remaining parameters use theoretically-grounded defaults: sparsity parameter *β* = *α*/15 to maintain appropriate sparsity relative to spatial constraints, orthogonality parameter *γ* = 1.5 × 10^−4^ × *p* (*p* is the number of genes) to address dimensional dependency of cosine similarity, radius parameter *λ* = 1.2 with adjustment guided by neighborhood connectivity analysis, and pattern number *r* = 30 for most datasets (with *r* = 40–50 recommended for highly heterogeneous samples). For the radius parameter, we provide visualization tools in our code to display the histogram of neighbor counts per spot after adjacency matrix construction, along with average neighbor count statistics. While we generally use the default value *λ* = 1.2, users should adjust *λ* when the average neighbor count falls outside the optimal range of 6–10 neighbors per spot to ensure appropriate spatial connectivity.

For downstream analyses, we set the threshold parameters as ϵ_*_ = 0.3 for pattern-specific gene detection and *ϵ* = 0.7 for spatially variable gene identification. These default thresholds were established through evaluation across multiple datasets by examining the respective score distributions (pattern contribution scores and reconstruction error rates) and validating spatial coherence of selected genes. Users can leverage our visualization tools to examine score histograms for natural separation points and assess spatial expression patterns of candidate genes at different thresholds. In spatial domain analysis, we recommend *N*_1_ = *r* − 5 − *n*_cluster_ and *N*_2_ = 3, where *n*_cluster_ represents the number of clusters. When the actual number of spatial domains is known, users should select *n*_cluster_ accordingly; otherwise, we recommend the default value of *n*_*c*_ = *n*_cluster_ = 8. Our sensitivity analysis (Supplementary Figs. 36–40) demonstrates that clustering performance remains stable when varying these parameters around their default values, supporting the robustness of our parameter selection strategy. While users retain flexibility to adjust parameters based on specific analytical needs, our default framework ensures reproducible results across diverse biological applications. Detailed parameter settings for all datasets analyzed in this study are provided in Supplementary Note 6 and Supplementary Table 1.

### Computational implementation

SEPAR is implemented in Python 3.8 using NumPy^73^, SciPy^74^, and scikit-learn^75^ for core numerical computations, with optional GPU acceleration via CuPy^76^ for processing large-scale spatial transcriptomics datasets. All computational analyses were performed on a high-performance computing cluster with specifications detailed in Supplementary Table 2.

### Competing methods

SEPAR was compared with state-of-the-art methods for different analytical tasks. For spatial domain identification and clustering analysis, we employed STAGATE^14^ (https://github.com/zhanglabtools/STAGATE), GraphST^15^ (https://github.com/JinmiaoChenLab/GraphST), and BASS^13^ (https://github.com/zhengli09/BASS) as competing methods. For spatially variable gene detection, we benchmarked against STAMarker^34^ (https://github.com/zhanglabtools/STAMarker), SPARK-X^33^ (https://xzhoulab.github.io/SPARK/), and SpatialDE^10^ (https://github.com/Teichlab/SpatialDE). For NMF based spatial methods, we benchmarked against SpiceMix^23^ (https://github.com/ma-compbio/SpiceMix), and NSFH^24^ (https://github.com/willtownes/nsf-paper). For spatial multi-omics integration, We compared with SpatialGlue^35^ (https://github.com/JinmiaoChenLab/SpatialGlue), MISO^36^ (https://github.com/kpcoleman/miso), spaVAE and spaMultiVAE^18^ (https://github.com/ttgump/spaVAE/).

To ensure fair comparison, all data were preprocessed according to the protocols described in the original publications of each competing method. The analyses were performed using the official implementations provided by the original authors, with default parameter settings as specified in their released versions.

### Statistics and reproducibility

This study analyzed datasets spanning diverse spatial scales to evaluate SEPAR’s broad applicability: small-scale targeted imaging (osmFISH: *n* = 4839 cells, 33 genes; MERFISH: *n* = 5488–5926 cells, 155 genes), medium-scale spatial transcriptomics (DLPFC: *n* = 3431–4788 spots per section, 12 sections; MISAR-seq: *n* = 1949 spots; CITE-seq: *n* = 2492 spots), and large-scale high-density profiling (Stereo-seq: *n* = 19,109 cells; VisiumHD: *n* = 545,872 spots; Xenium: *n* = 340,837 cells). For statistical comparisons, sample sizes were determined by biological relevance: marker validation used known layer-specific genes (*n* = 27 identified vs n = 39 missing); method comparisons used uniquely detected SVGs (*n* = 228–986 genes per method); expression distribution analyses used n = 33–5,000 genes depending on available features. Pattern-specific gene sets ranged from 1–1,210 genes, with median 69–152 genes per pattern, reflecting natural variation in spatial expression complexity. Specific sample sizes for individual analyses are provided in figure legends.

Non-parametric tests were used throughout. Mann-Whitney U tests (one-sided) were used to compare Moran’s I^48^ distributions between method-specific SVGs and background genes, and to compare spatial autocorrelation between SEPAR-identified canonical layer markers versus non-identified markers. Cell-type enrichment used CellGO^44^ with Fisher’s exact test. Gene ontology enrichment used g:Profiler^46^ with hypergeometric test and g:SCS multiple testing correction. Motif enrichment used HOMER^65^ with hypergeometric test and Benjamini-Hochberg FDR correction. Peak-gene associations were annotated using ChIPseeker^64^ with default parameters. DEGs between cortical layers were identified using DESeq2^49^ with Wald test and Benjamini-Hochberg FDR correction. For all analyses, *p*-value  < 0.05 was considered statistically significant unless otherwise specified. Reproducibility was validated by repeating experiments with different initial values.

### Ethics statement

All the datasets used in this study are publicly available. All of the original studies have obtained appropriate ethical approval and informed consent as documented in the respective source publications^1,11,12,40–43^.

## Supplementary information

Supplementary Information

Description of Additional Supplementary files

Supplementary Data 1

Article File-Pdf

## Data Availability

The spatial transcriptomics datasets analyzed in this study are publicly available. Source data underlying figures are available in Supplementary Data 1: the human DLPFC dataset^1^ can be accessed at spatialLIBD project website (http://research.libd.org/spatialLIBD/); the processed mouse olfactory bulb Stereo-seq data^40^ is available at 10.5281/zenodo.8356092; the MERFISH data^42^ can be downloaded from 10.5061/dryad.8t8s248; the osmFISH data^41^ is available at http://linnarssonlab.org/osmFISH/availability/; the MISAR-seq^12^ and spatial CITE-seq^11^ datasets are available at 10.5281/zenodo.7480069and https://www.ncbi.nlm.nih.gov/geo/query/acc.cgi?acc=GSE213264, respectively. The human colorectal cancer (CRC) datasets including VisiumHD, Visium, and Xenium data for P2CRC samples^43^ are available at the following website: https://www.10xgenomics.com/products/visium-hd-spatial-gene-expression/dataset-human-crc.

## Code availability

The SEPAR software package is freely available at https://github.com/zerovain/SEPAR^77^ under the MIT license. Detailed documentation and tutorials are available at https://SEPAR.readthedocs.io.
