# Supplementary material for: SEPAR enables spatial metagene discovery and associated molecular pattern characterization in spatial transcriptomics and multi-omics datasets
Source: Commun Biol. 2025 Dec 10;9:77. doi: 10.1038/s42003-025-09340-w (PMC12820152; doi:10.1038/s42003-025-09340-w)
Supplement: Supplementary file 1 — Supplementary Information [file 42003_2025_9340_MOESM1_ESM.pdf]

Supplementary Information for "SEPAR enables spatial metagene discovery and associated molecular pattern characterization in spatial transcriptomics and multi-omics datasets"

November 21, 2025

Supplementary Notes

Supplementary Note 1: SEPAR performance in heterogeneous colorectal cancer tissue

To evaluate SEPAR's performance in heterogeneous disease tissues without clear structural layering, we applied it to a 10× colorectal cancer VisiumHD dataset containing over 500,000 spots [1]. SEPAR demonstrated excellent computational efficiency, completing the analysis within 1000 seconds and exhibiting superior scalability compared to other NMF-based methods (Supplementary Fig. 32).

SEPAR successfully identified 12 distinct spatial patterns in the colorectal cancer tissue (Supplementary Fig. 33a). The patterns exhibited varying pattern-specific gene numbers, with Pattern 1 containing 1023 genes, Pattern 3 containing 691 genes, and others ranging from 1 to 249 genes.

Functional enrichment analysis revealed distinct cancer-associated processes for all identified patterns (Supplementary Fig. 33b). Pattern 1 showed enrichment for high-energy metabolic processes, including oxidative phosphorylation, thermogenesis, cell cycle regulation, and neurodegenerative disease-associated gene sets capturing mitochondrial and proteostasis modules, suggesting metabolically hyperactive proliferative zones. Pattern 3 exhibited enrichment for the Wnt signaling pathway, tight junctions, the Hippo signaling pathway, and vascular transport processes, indicating regions maintaining epithelial architecture and regulatory mechanisms. Additional patterns captured diverse cancer-related processes (Supplementary Fig. 34): Pattern 4 was characterized by extracellular matrix organization and collagen remodeling; Pattern 6 showed inflammatory pathway enrichment, including IL-17 and TNF signaling; Pattern 8 demonstrated angiogenesis regulation and blood vessel morphogenesis, representing key processes in tumor progression.

High-resolution spatial visualization demonstrates that Patterns 1 and 3 represent functionally distinct compartments with complementary spatial organization (Supplementary Fig. 33c). Pattern 1 shows concentrated distribution in specific tumor regions with high metabolic activity, while Pattern 3 displays complementary spatial localization in epithelial regulatory zones. The difference map highlights their opposing spatial gradients, indicating an organization in which metabolically hyperactive proliferative zones are adjacent to, yet functionally distinct from, signaling-regulatory regions.

The spatial clustering analysis demonstrates SEPAR's ability to partition complex cancer tissue into coherent spatial domains (Supplementary Fig. 33d). This reveals delineation of functionally distinct tissue regions, demonstrating SEPAR's capability to resolve spatial organization in heterogeneous disease tissue. This application demonstrates SEPAR's robust performance in discovering biologically meaningful spatial domains in complex disease tissues, extending its applicability beyond well-structured contexts to clinically relevant pathological conditions.

Supplementary Note 2: SEPAR analysis of MERFISH data

To further validate the performance of SEPAR, we analyzed a MERFISH [2] dataset from the mouse hypothalamic preoptic region [3], which measured the expression of 155 genes across 64,373 cells. This region is essential for regulating parental and mating behaviors. We applied SEPAR to identify the spatial metagene expression patterns and their associated pattern-specific genes.

SEPAR identified 30 distinct spatial metagene patterns (Supplementary Fig. 17). We directly used the spatial patterns extracted by SEPAR as low-dimensional representations for clustering. As shown in Supplementary Fig. 18a and Supplementary Fig. 18b, the computation of SEPAR is much faster than BASS, though the spatial domain identification accuracy of SEPAR ranks second among four methods, just behind BASS. Given that the ground truth annotations were originally provided by BASS [4], SEPAR's competitive performance (ARI = 0.454, NMI = 0.565) demonstrates its robust capability in spatial domain identification. Notably, when comparing with Allen Reference Atlas – Mouse Brain [5] (Supplementary Fig. 19), SEPAR's identification of the medial preoptic nucleus (MPN) actually showed better anatomical correspondence than BASS, suggesting its biological relevance. SEPAR costs only 9.13 seconds for the computation, while BASS takes 508.77 seconds. In contrast, STAGATE and GraphST reached lower spatial domain identification accuracy with more computational time (STAGATE: 43.03s, GraphST: 21.73s). SEPAR demonstrates the highest computational efficiency, balancing efficacy and efficiency among the four methods.

We also conducted gene expression refinement and spatial gene co-expression analysis on this dataset. After the refinement, the spatial correlation of gene expression is considerably improved (Supplementary Fig. 18c). Supplementary Fig. 18d displays the expression levels of 6 genes before and after refinement, showing enhanced spatial patterns. To validate the biological authenticity of these refined patterns, we examined their consistency across five independent adjacent tissue slices from the same MERFISH hypothalamus dataset (Supplementary Fig. 20). The analysis revealed that SEPAR consistently recovered similar spatial patterns for representative genes such as *Gal* and *Trh* across all adjacent slices, demonstrating that the refined expression patterns reflect genuine biological structures rather than method-specific artifacts. Additionally, comparative analysis with spaVAE [6] showed that SEPAR exhibits reduced hallucination artifacts for most genes, though both methods face challenges for extremely sparse genes like *Ebf3* due to the inherent difficulty of refining patterns from minimal raw signal (Supplementary Fig. 21).

Supplementary Fig. 18e presents a co-expression heatmap of the top 30 highly variable genes before and after refinement, showing 31 pairs with strong correlations (correlation coefficient > 0.5) after refinement, whereas only 15 correlations were observed in the raw data. The refined data not only exhibited correlation improvement of known correlated genes but also identified new correlated gene pairs. For instance, *Gal* and *Trh* were not recognized as co-expressed genes in the raw data, while they showed highly correlated expression patterns in similar domains after refinement, with corresponding cells being spatially proximate, as previously shown in Supplementary Fig. 18d. Similarly, *Ebf3*, *Ermn*, *Opalin*, and *Sgk1*, whose spatial expression patterns are visualized in Supplementary Fig. 18d, were identified as correlated gene pairs after refinement, with their high-expression cells exhibiting clear spatial adjacency in the tissue. This illustrates that gene refinement using SEPAR enables spatial gene co-expression analysis, leveraging spatial location information.

Supplementary Note 3: Computational efficiency validation

Emerging high-resolution spatial technologies like VisiumHD and Xenium generate datasets comprising millions of spots, raising computational scalability concerns. To assess SEPAR's performance under such conditions, we applied it to a 10× colorectal cancer VisiumHD dataset containing over 500,000 spots [1], where SEPAR completed the analysis within 1,000 seconds.

By subsampling this dataset, we demonstrated that its runtime scales linearly with the number of spots (Supplementary Fig. 32a). Comparative analysis across the 12 slices from the DLPFC dataset shows superior efficiency compared to other NMF-based methods—over 10 times faster than NSFH and over 100 times faster than SpiceMix (Supplementary Fig. 32b). These experiments demonstrate SEPAR's practical applicability to large-scale spatial datasets generated by current high-resolution technologies.

All computational analyses were performed on a high-performance computing cluster with specifications detailed in Supplementary Table 2. SEPAR was implemented in Python 3.8 using NumPy [7], SciPy [8], and scikit-learn [9] for core numerical computations, with optional GPU acceleration via CuPy [10] for processing large-scale spatial transcriptomics datasets.

Supplementary Note 4: Expression refinement validation

While SEPAR's expression refinement demonstrates robust performance, potential concerns about over-smoothing warrant validation. Consistency analysis across datasets indicated reproducible pattern recovery with reduced artifacts compared to alternative methods (Supplementary Figs. 21, 25).

To provide independent validation, we conducted cross-technology comparison using adjacent colorectal cancer tissue sections, where SEPAR-refined Visium data showed enhanced concordance with high-resolution Xenium patterns [1] (Supplementary Fig. 35). These validations provide evidence for the biological validity of our refinement approach, while acknowledging challenges for extremely sparse genes.

Detection of very thin anatomical structures remains challenging due to current spatial transcriptomics resolution limit and the difficulty of aligning gene expression boundaries with morphological annotations. This challenge is shared across spatial analysis methods, as recent comprehensive benchmarking on the DLPFC 151507 slice shows no spatial clustering method achieves clustering ARI above 0.55 against manual annotations [11], indicating the inherent difficulty of reproducing morphological boundaries through expression-based computational methods.

For multi-omics SRT data, a fundamental limitation shared by computational methods is the inability to establish causality from spatial co-localization. While SEPAR identifies spatially coherent patterns across molecular modalities, spatial co-localization represents correlation rather than causation. True validation requires experimental follow-up including temporal profiling, perturbation experiments, and functional assays to validate proposed molecular interactions.

Supplementary Note 5: Computational algorithm for SEPAR

The SEPAR algorithm solves the optimization problem in Equation (1) of the main text through iterative multiplicative updates. Below we present the detailed computational procedure.

Algorithm 1 Spatial gene expression pattern recognition algorithm (SEPAR)

Require: Non-negative data matrix X and parameters r, α, β, and γ
Ensure: Basis matrix W, coefficient matrix H
Initialize non-negative matrices W and H using standard NMF algorithm
repeat
  Calculate PSS for each pattern as defined in Equation (4) of the main text
  for each element W_{ij} **do**
    $W_{ij} \leftarrow W_{ij} \cdot \frac{(XH^T)_{ij}+\alpha PSS_j(AW)_{ij}}{(WHH^T)_{ij}+\alpha PSS_j(DW)_{ij}+\beta}$
  end for
  for each element H_{ij} **do**
    $H_{ij} \leftarrow H_{ij} \cdot \frac{(W^TX)_{ij}}{(W^TWH)_{ij}+\gamma\sum_{k\neq i}\langle h_i,h_k\rangle H_{kj}}$
  end for
  for each row i in H **do**
    $norm \leftarrow \sqrt{\sum_{j=1}^{p} H_{ij}^2}$
    if $norm > 0$ **then**
      $H_{i:} \leftarrow H_{i:}/norm$
      $W_{:i} \leftarrow W_{:i} \cdot norm$
    end if
  end for
  Ensure non-negativity: $W \leftarrow \max(W, 0)$, $H \leftarrow \max(H, 0)$
until convergence or maximum iterations reached

Supplementary Note 6: Parameter settings for different datasets

Hyperparameter settings used for SEPAR analysis across all datasets are provided in Supplementary Table 1. For the DLPFC dataset, we used consistent parameters ($\alpha = 0.3$, $\beta = 0.02$, $\gamma = 0.3$) across individual slices, with adjusted nearest neighbor parameters (N_1, N_2) to account for variations in tissue density. The multislice analysis used $\alpha = 0.5$, $\beta = 0.003$, $\gamma = 0.5$ to balance information sharing across slices. For other datasets, parameters were tuned to account for technology-specific characteristics such as cell/spot density and gene coverage.

Supplementary Tables

Supplementary Table 1: Hyperparameter settings for SEPAR across different spatial datasets and experimental conditions

Dataset	Slice	r	α	β	γ	N_1	N_2
DLPFC	151507	30	0.3	0.02	0.3	16	5
	151508	30	0.3	0.02	0.3	16	5
	151509	30	0.3	0.02	0.3	16	5
	151510	30	0.3	0.02	0.3	16	5
	151669	30	0.3	0.02	0.3	18	5
	151670	30	0.3	0.02	0.3	18	5
	151671	30	0.3	0.02	0.3	18	5
	151672	30	0.3	0.02	0.3	18	5
	151673	30	0.3	0.02	0.3	16	5
	151674	30	0.3	0.02	0.3	16	5
	151675	30	0.3	0.02	0.3	16	5
	151676	30	0.3	0.02	0.3	16	5
	multi-slice	30	0.5	0.003	0.5	13	5
Stereo-seq	–	30	0.8	0.05	0.5	16	4
osmFISH	–	30	1.0	0.05	0.01	0	0
MERFISH	–	30	1.0	0.05	0.01	5	7
CITE-seq	–	30	0.5	0.01	0.5	18	7
MISAR-seq	–	30	0.5	0.01	0.5	15	1

Supplementary Table 2: Hardware configuration for computational analyses

**Component**	**Specification**
CPU	Intel Xeon Gold 6226R (2.90 GHz, 16 cores per job)
GPU	NVIDIA A30 (24GB GDDR6, CUDA 12.2)
Memory	1TB total RAM with dynamic allocation
Storage	High-speed SSD
OS & Scheduler	Ubuntu 22.04.4 LTS with SLURM

Supplementary Figures

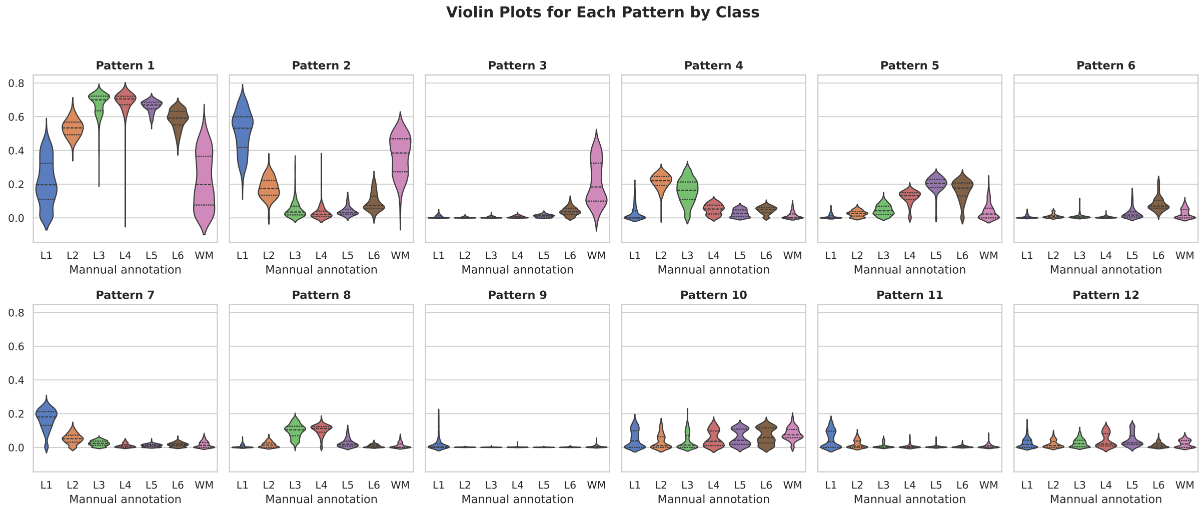

Supplementary Figure 1: The distribution of expression levels for each pattern illustrated using violin plots across seven manually annotated regions (L1: n=817, L2: n=305, L3: n=1,215, L4: n=369, L5: n=675, L6: n=486, WM: n=354 spots). Violin plots show median (center line), interquartile range (inner lines), and full data distribution (violin shape).

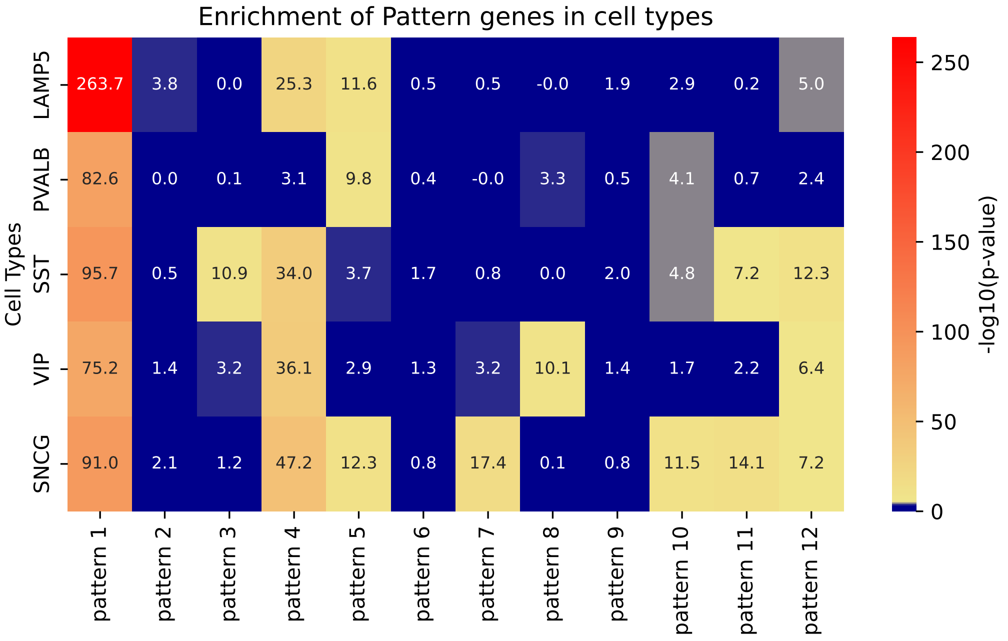

Supplementary Figure 2: Cell-type enrichment analysis of 12 pattern-specific gene sets using CellGO on DLPFC section 151507 (n=4,221 spots; gene set sizes: 7-1,210 genes). Analysis focused on inhibitory interneuron (InN) cell types including LAMP5, PVALB, SST, VIP, and SNCG subtypes.

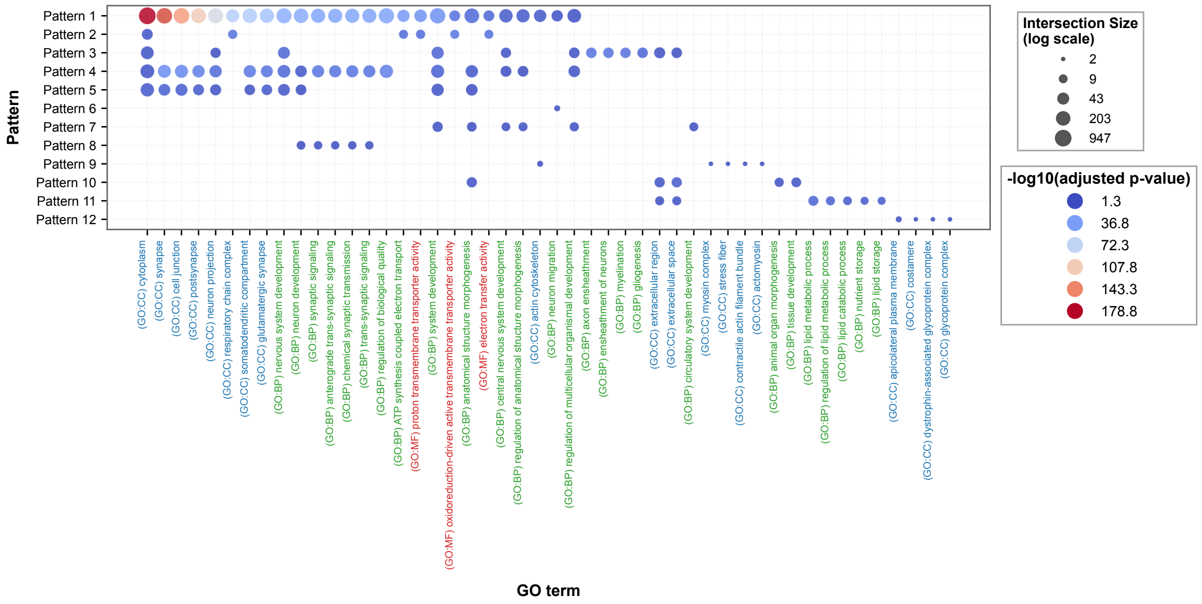

Supplementary Figure 3: Gene Ontology enrichment analysis for DLPFC pattern-specific genes. Bubble plot shows the union of the top five significantly enriched GO terms from each of the 12 pattern-specific gene sets identified in DLPFC section 151507 (gene set sizes: 7–1,210 genes). Each bubble represents a GO term that is significantly enriched in a given pattern (adjusted p-value < 0.05, Fisher's exact test with Benjamini-Hochberg correction). The absence of a bubble indicates that the term is not significantly enriched in that pattern. Bubble size represents the intersection size (number of genes from the pattern-specific set annotated to the GO term) and is log-scaled for visual clarity. Bubble color indicates enrichment significance as $-\log_{10}$(adjusted p-value), with warmer colors (red) denoting higher significance. GO categories are color-coded in x-axis labels: Biological Process (GO:BP, green), Molecular Function (GO:MF, red), and Cellular Component (GO:CC, blue).

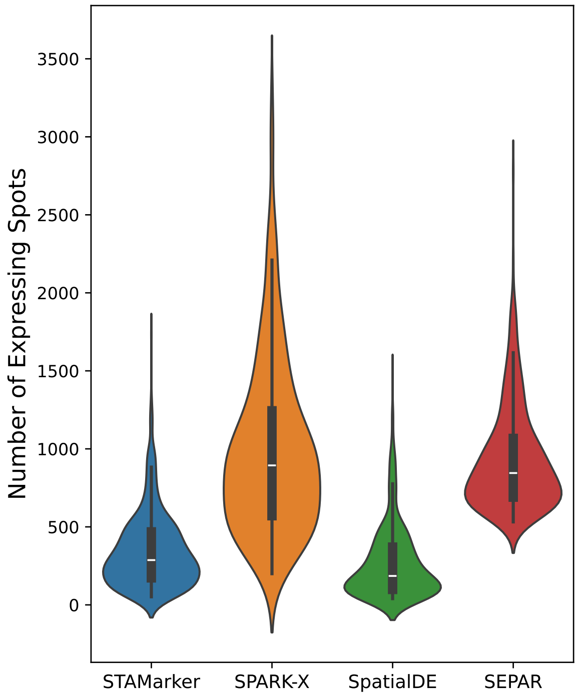

Supplementary Figure 4: Violin plot of expressing spot number of uniquely identified SVGs for each method on DLPFC dataset [12] (SEPAR only: n=399 genes, SpatialDE only: n=456 genes, SPARK-X only: n=228 genes, STAMarker only: n=579 genes). Violin plots show median (center line), interquartile range (box), and full data distribution (violin shape).

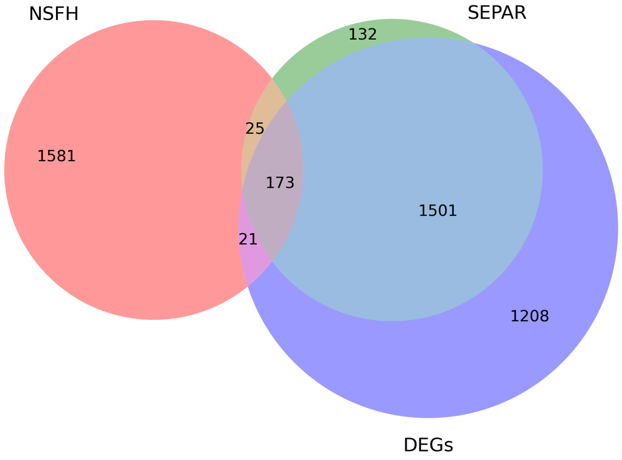

Supplementary Figure 5: Venn plot comparing the SVGs from SEPAR and NSFH and the layer-specific DEGs selected by DESeq2 on DLPFC section 151507 (n=4,221 spots). Gene counts shown in diagram.

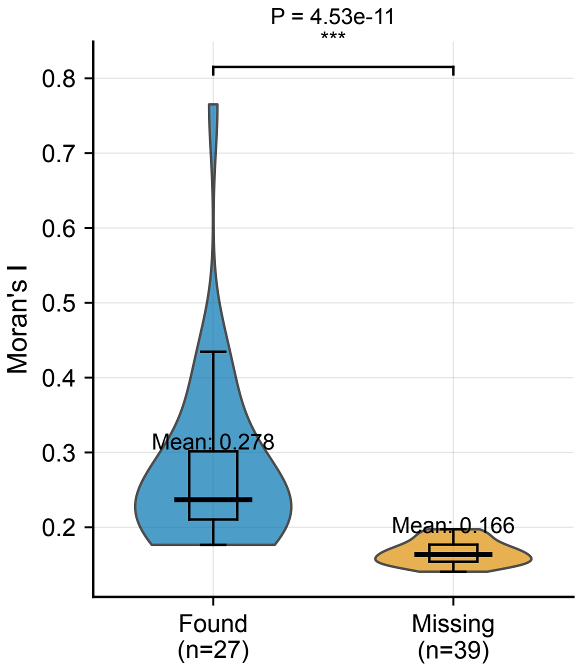

Supplementary Figure 6: **Validation of SEPAR's SVG specificity using known layer-specific markers.** Comparison of Moran's I values between layer-specific markers found by SEPAR (n=27) versus missing markers (n=39). Missing markers show significantly lower spatial autocorrelation (one-sided Mann-Whitney U test, p = 4.53e-11), validating SEPAR's selectivity. Violin plots display median (center line), quartiles (box), and whiskers (1.5×IQR).

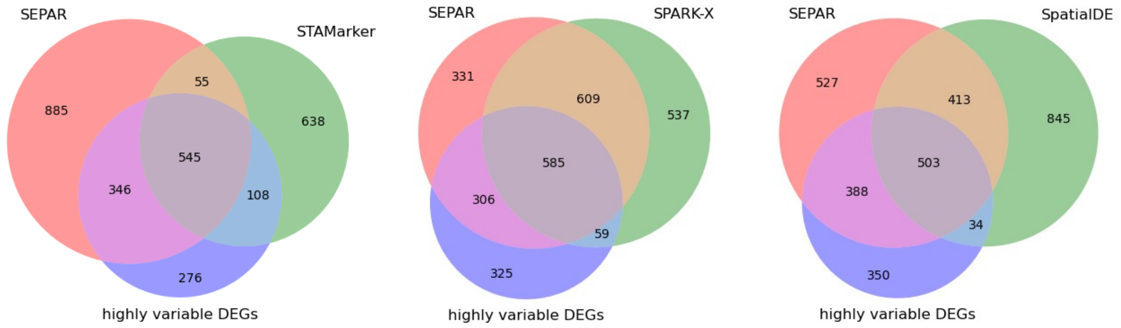

Supplementary Figure 7: The SEPAR analysis conducted on section 151507 of the DLPFC dataset [12]. A Venn diagram compares SEPAR with STAMarker, SPARK-X, and SpatialDE for high variable SVG identification on DLPFC section 151507 (n=4,221 spots). Gene counts shown in diagram.

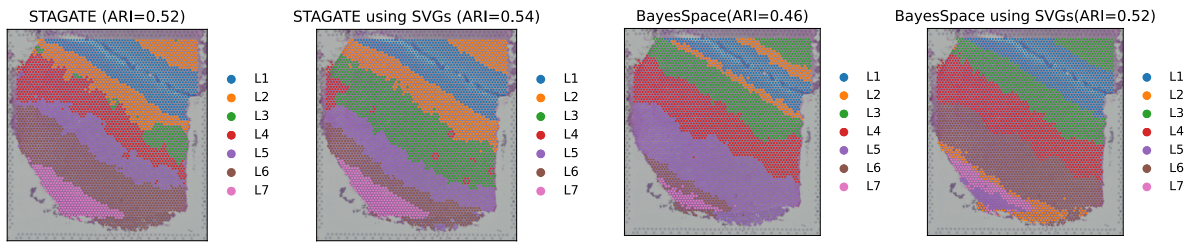

Supplementary Figure 8: Clustering results of STAGATE and BayesSpace on DLPFC section 151507 (n=4,221 spots) using their default settings (top 3000 HVGs for STAGATE, top 2000 HVGs for BayesSpace) or using SVGs selected by SEPAR.

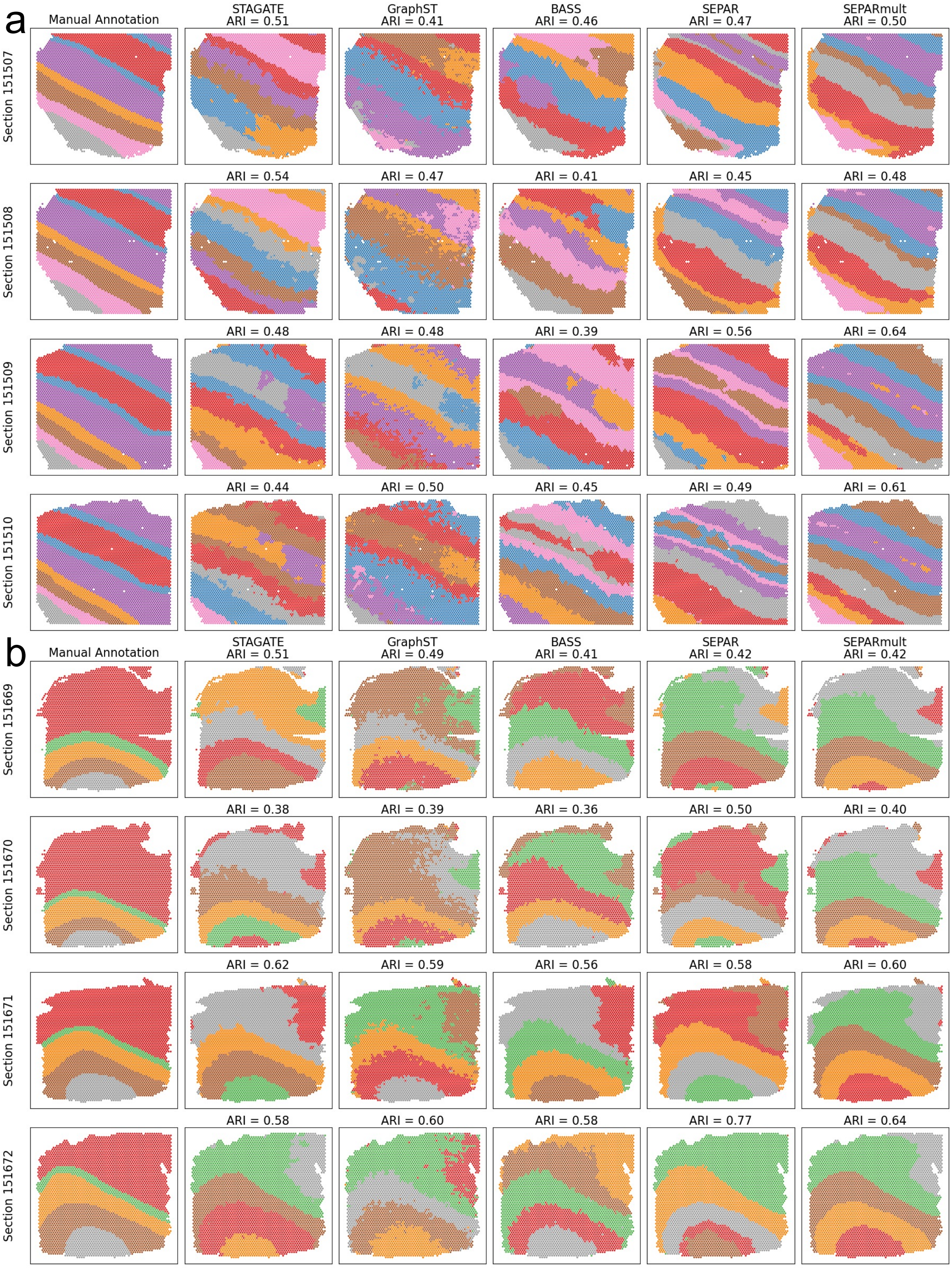

Supplementary Figure 9: **Spatial domain identification comparison across DLPFC sections using multiple methods.** (**a**) Sections 151507-151510 (n=4,221; 4,381; 4,788; 4,595 spots respectively) display manual annotations along with cluster identifications using STAGATE, GraphST, BASS, SEPAR, and SEPARmult. (**b**) Sections 151669-151672 (n=3,636; 3,484; 4,093; 3,888 spots respectively) are analyzed for manual annotations and cluster identifications using the same methods.

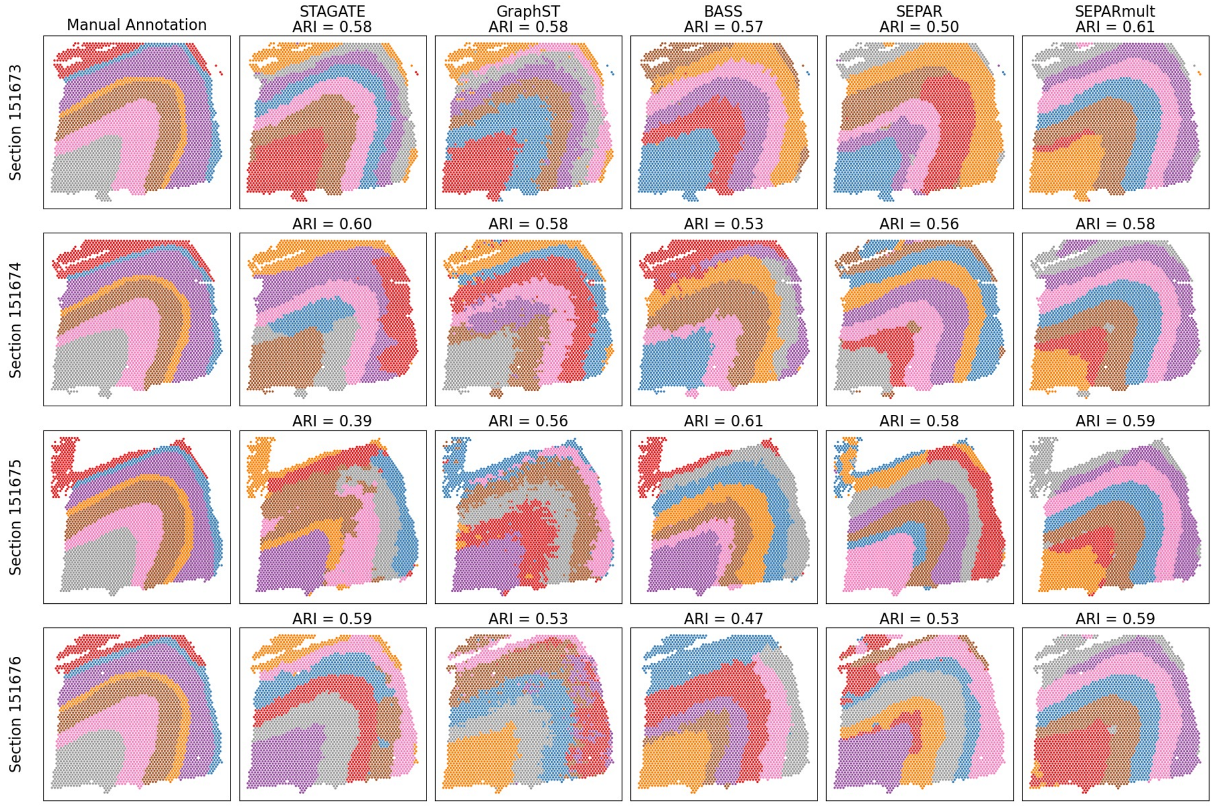

Supplementary Figure 10: Sections 151673-151676 of the DLPFC dataset (n=3,611; 3,635; 3,566; 3,431 spots respectively) display manual annotations along with cluster identifications using STAGATE, GraphST, BASS, SEPAR, and SEPARmult.

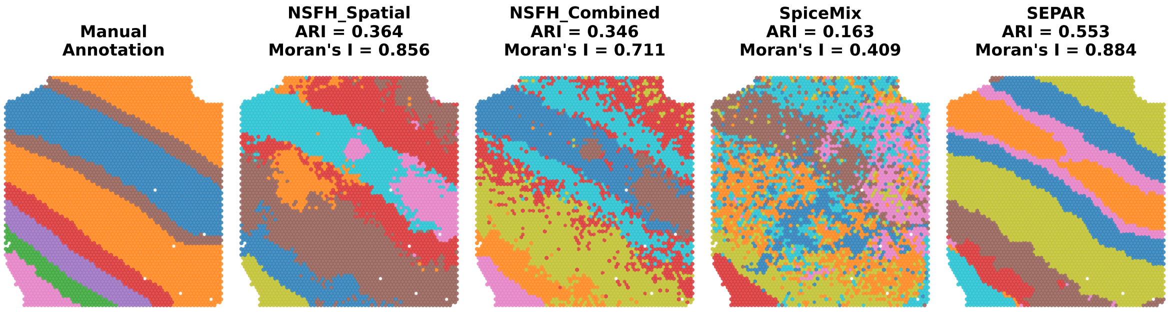

Supplementary Figure 11: Spatial clustering performance comparison on DLPFC section 151509 (n=4,788 spots). SEPAR achieves superior clustering accuracy (ARI = 0.553) and spatial coherence (Moran's I = 0.884) compared to NSFH using only spatial components (ARI = 0.364), NSFH using combined spatial and nonspatial components (ARI = 0.346), and SpiceMix (ARI = 0.163).

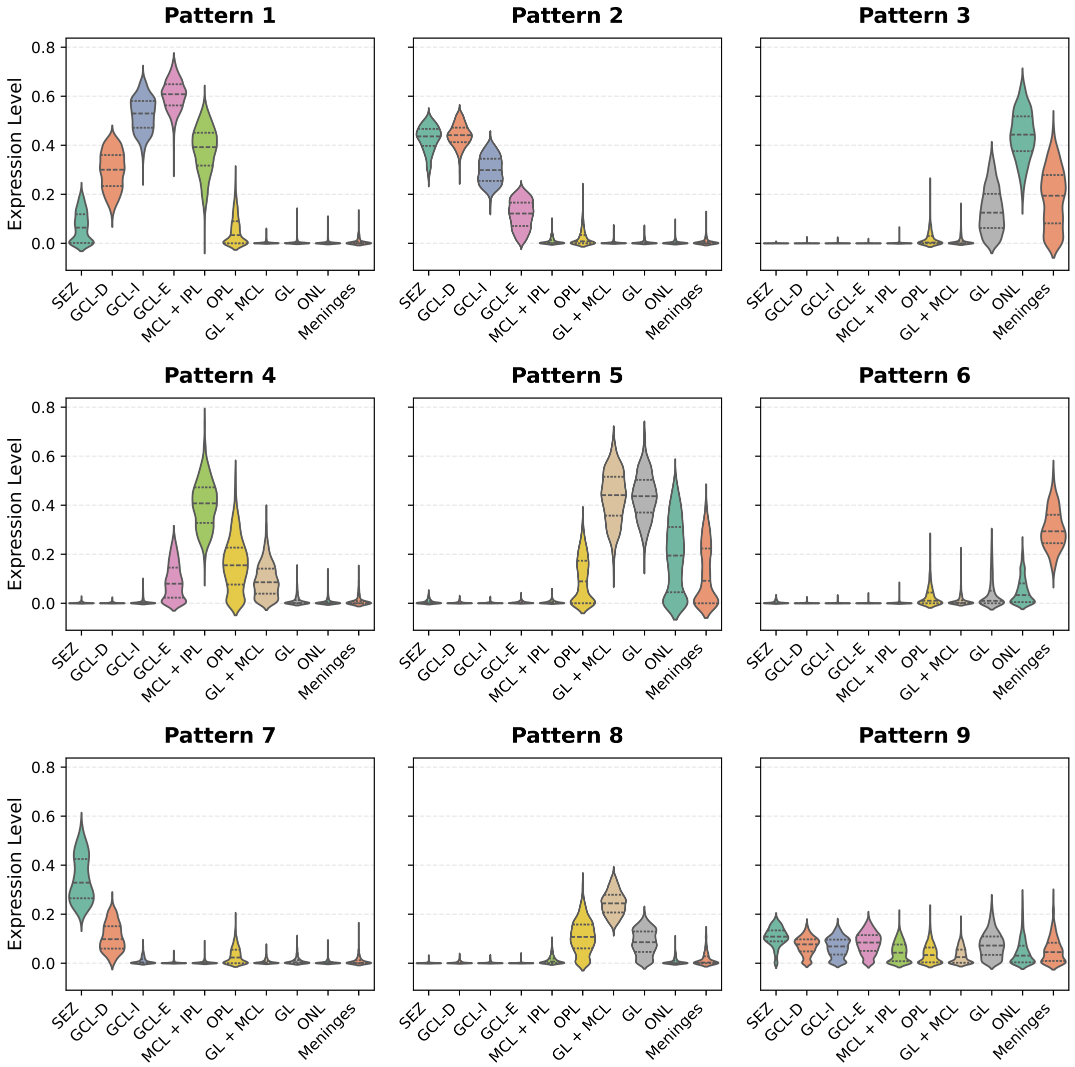

Supplementary Figure 12: Expression distribution of the 9 most significant SEPAR-identified patterns across ten annotated olfactory bulb layers in the Stereo-seq dataset (Meninges: n=1,029, ONL: n=1,655, GL: n=1,800, GL+MCL: n=2,670, OPL: n=1,786, MCL+IPL: n=2,123, GCL-E: n=2,663, GCL-I: n=2,658, GCL-D: n=1,922, SEZ: n=803 spots). Violin plots show median (center line), interquartile range (box), and full data distribution (violin shape).

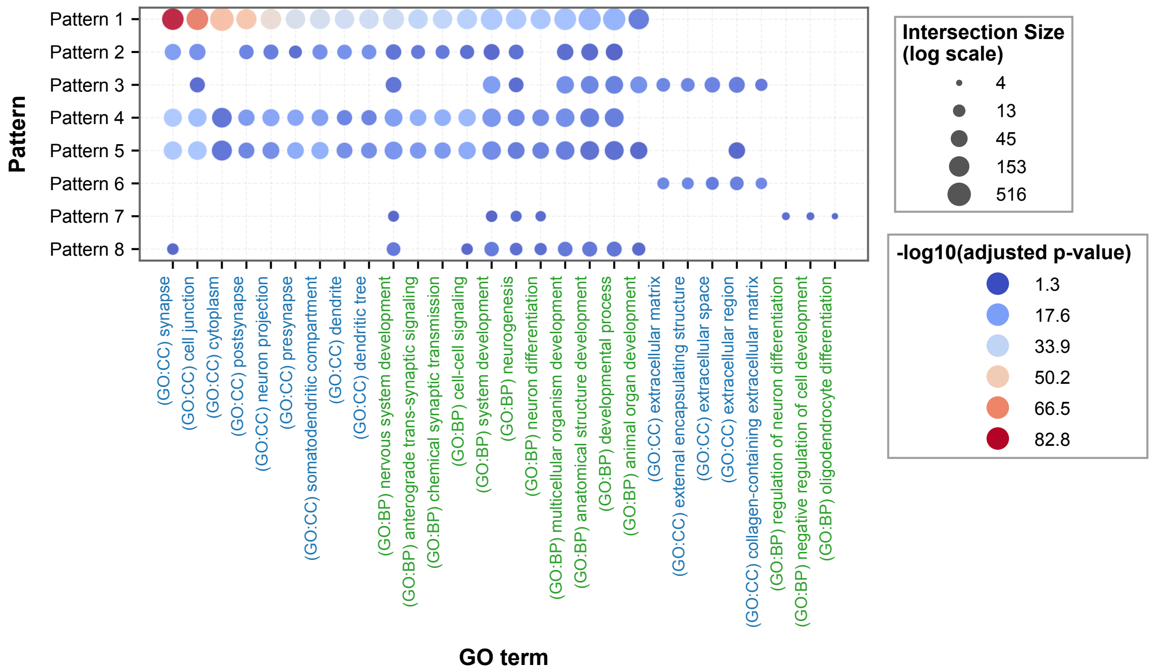

Supplementary Figure 13: Gene Ontology enrichment analysis for Stereo-seq pattern-specific genes. Bubble plot shows the union of the top five significantly enriched GO terms from each of the 9 pattern-specific gene sets (gene set sizes: 13–753 genes). Each bubble represents a GO term that is significantly enriched in a given pattern (adjusted p-value < 0.05). Pattern 9 shows no bubbles because no GO terms were significantly enriched for this pattern. Bubble size represents the intersection size (number of genes annotated to the term) and is log-scaled. Bubble color indicates enrichment significance as $-\log_{10}$(adjusted p-value). GO categories are color-coded in x-axis labels: GO:BP (green), GO:MF (red), and GO:CC (blue).

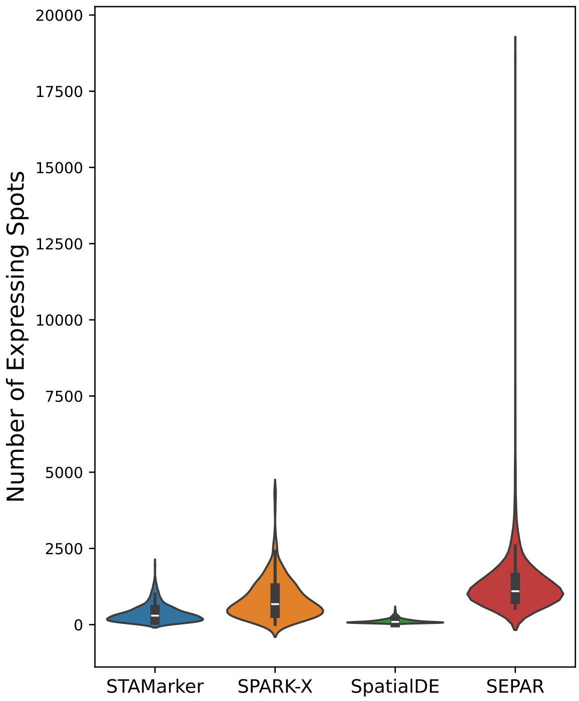

Supplementary Figure 14: Violin plot of expressing cell number of uniquely identified SVGs for each method on Stereo-seq dataset (SEPAR only: n=402 genes, SpatialDE only: n=986 genes, SPARK-X only: n=311 genes, STAMarker only: n=858 genes). Violin plots show median (center line), interquartile range (box), and full data distribution (violin shape).

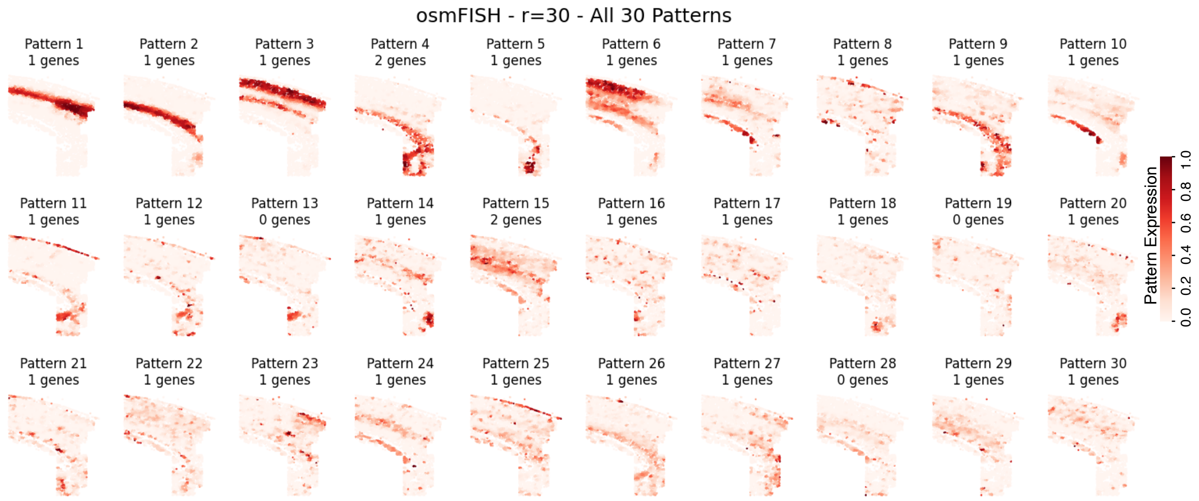

Supplementary Figure 15: Thirty spatial metagene patterns identified by SEPAR on osmFISH dataset (n=4,839 cells, r=30).

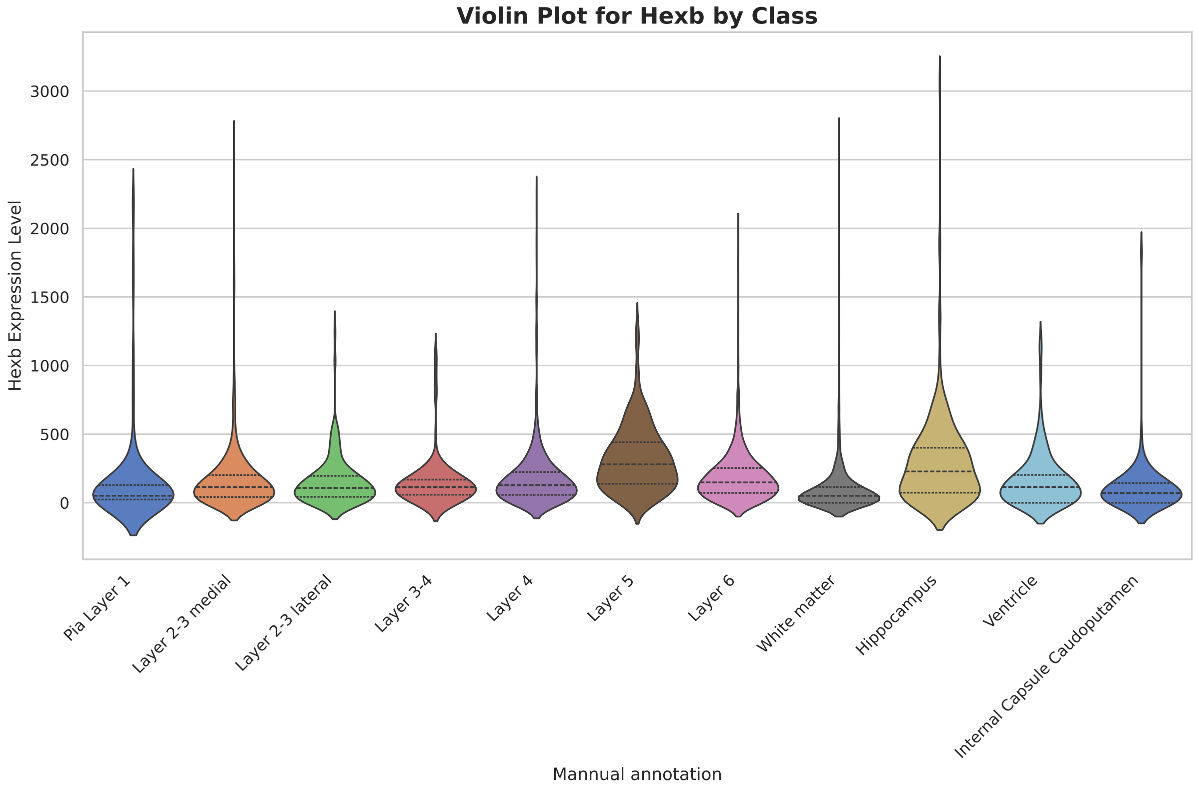

Supplementary Figure 16: Violin plots compare the expression distribution of the Hexb gene across 11 manually annotated domains in the osmFISH dataset (Pia Layer 1: n=159, Layer 2-3 medial: n=549, Layer 2-3 lateral: n=254, Layer 3-4: n=131, Layer 4: n=1,002, Layer 5: n=295, Layer 6: n=1,015, White matter: n=877, Hippocampus: n=322, Ventricle: n=129, Internal Capsule Caudoputamen: n=106 cells). Violin plots show median (center line), interquartile range (inner lines), and full data distribution (violin shape).

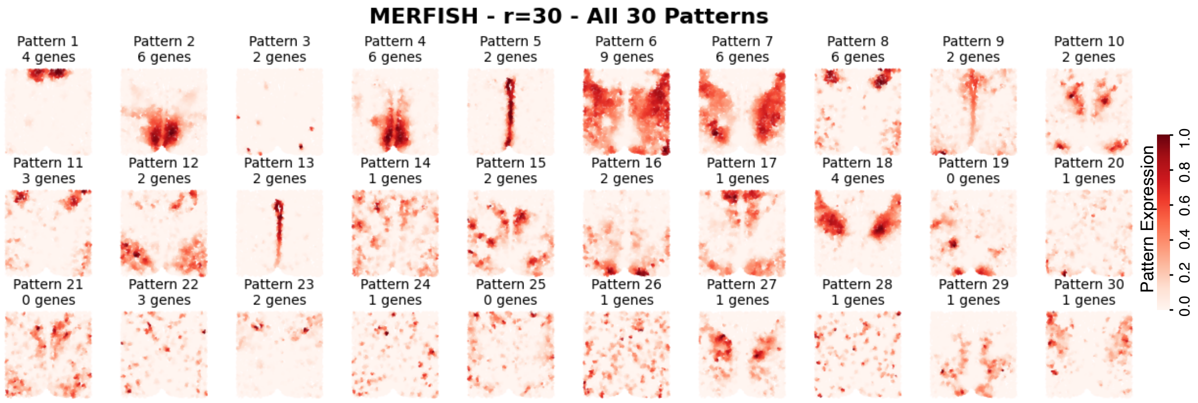

Supplementary Figure 17: Thirty spatial metagene patterns identified by SEPAR on MERFISH dataset (n=5,926 cells, r=30).

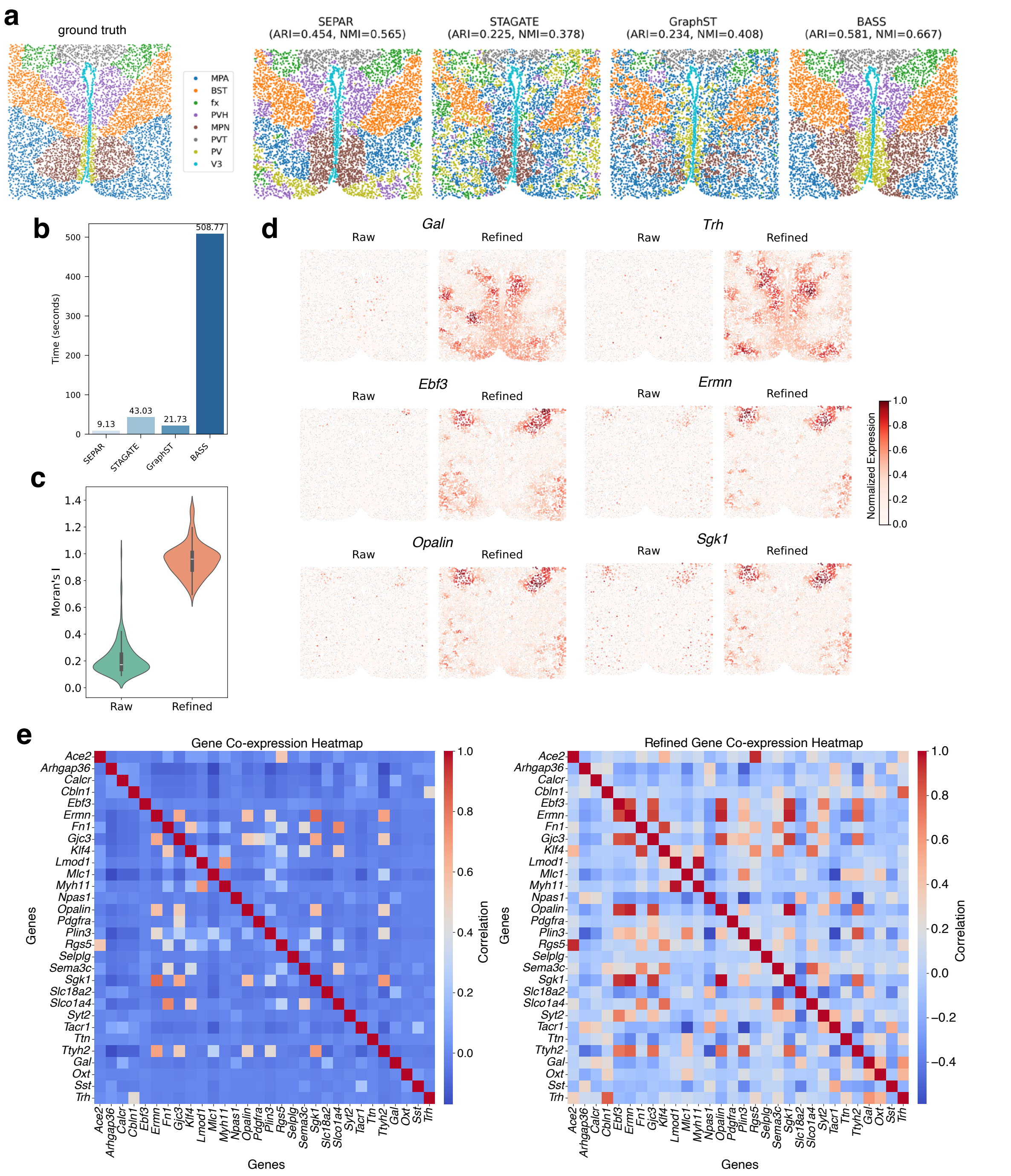

Supplementary Figure 18: **Analysis of the mouse hypothalamic preoptic region generated by MERFISH [2].** (**a**) Spatial domain identification using SEPAR, STAGATE, GraphST and BASS. The ground truth annotation was obtained from Ref. [4]. (**b**) Comparison of running time for SEPAR, STAGATE, GraphST and BASS. (**c**) Violin plots of Moran's I values for gene expression before and after refinement(n=155 genes per condition). (**d**) Visualization of 6 genes before and after gene expression refinement. Expression values are normalized to [0,1] range within each panel for visualization. (**e**) Gene co-expression heatmaps generated from raw (left) and refined (right) gene expression. Violin plots show median (center line), interquartile range (box), and full data distribution (violin shape).

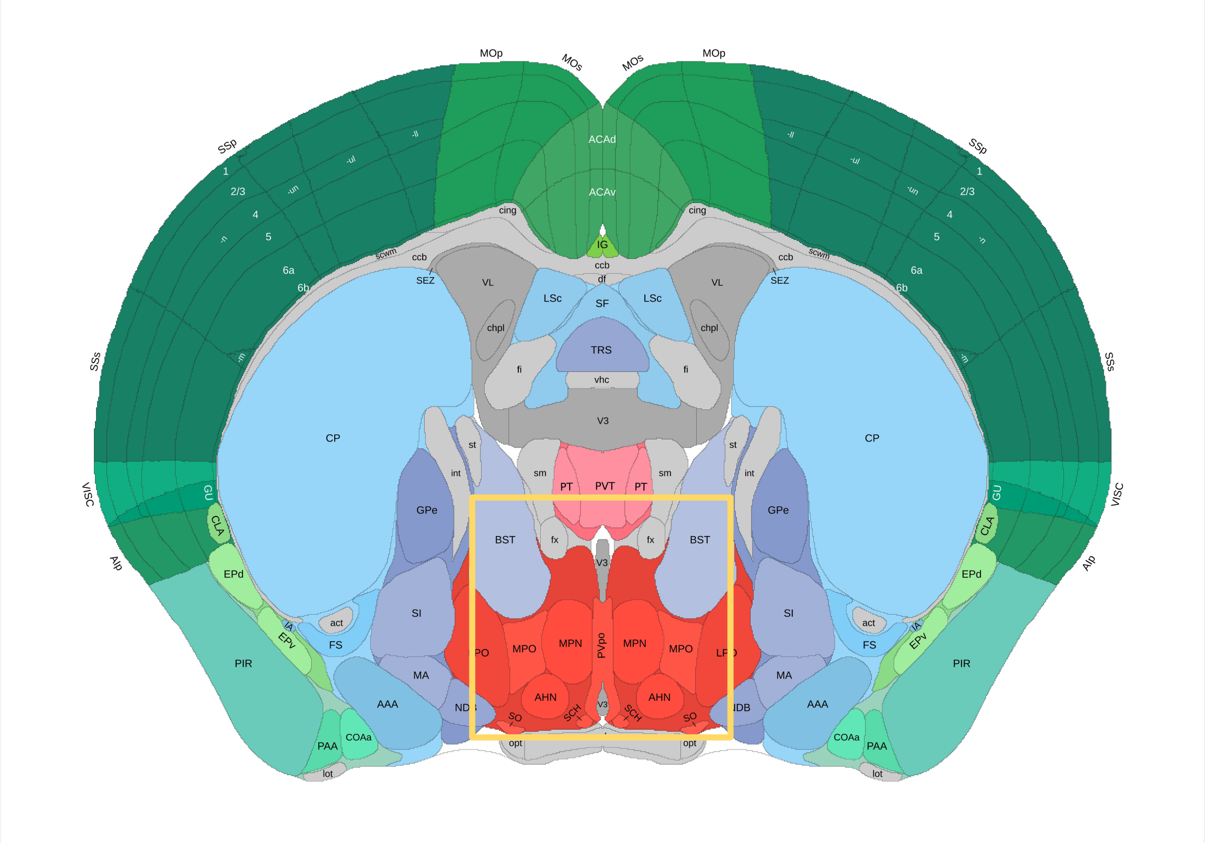

Supplementary Figure 19: Anatomical reference showing mouse brain structures from the Allen Reference Atlas – Mouse Brain [5], with the yellow box delineating the hypothalamic preoptic region that corresponds to the MERFISH-profiled area.

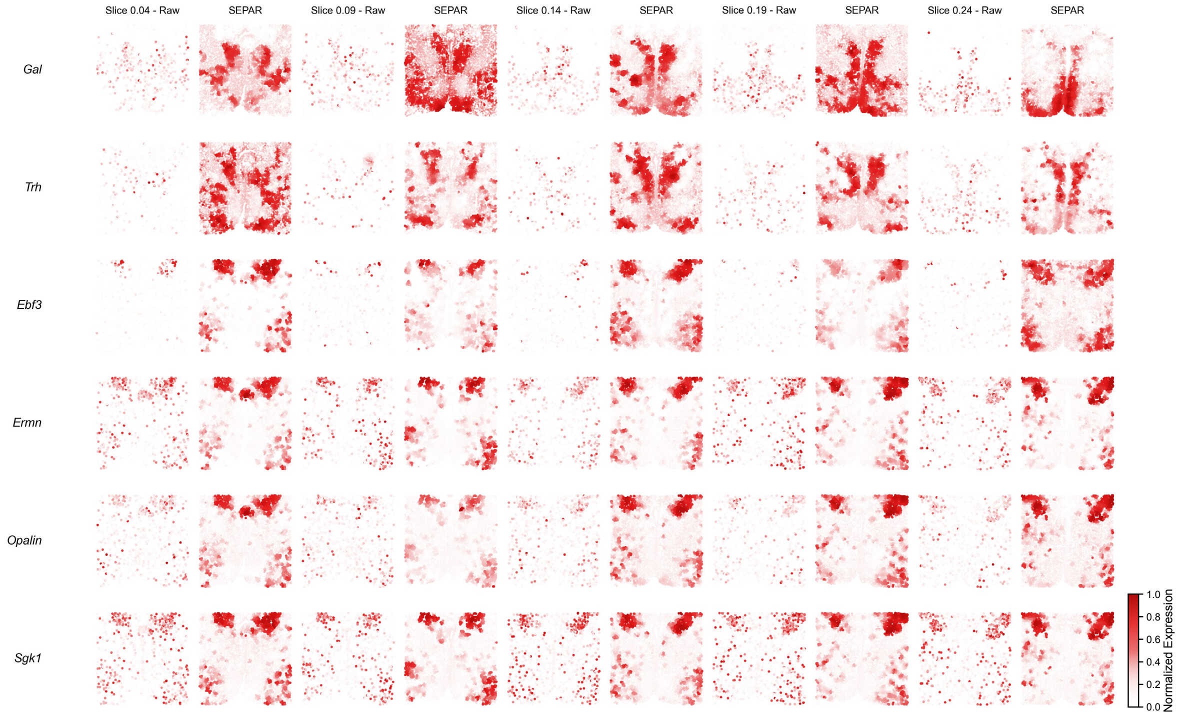

Supplementary Figure 20: Validation of SEPAR's expression refinement using five adjacent tissue slices from MERFISH mouse hypothalamus dataset [2] (slices 0.04, 0.09, 0.14, 0.19, 0.24 with n=5,488; 5,557; 5,926; 5,803; 5,543 cells respectively). For each of six representative genes, raw expression (Raw) and SEPAR-refined expression (SEPAR) are shown across slices. Expression values are normalized to [0,1] range within each panel for visualization. Pattern consistency indicates SEPAR robustly captures biological structures.

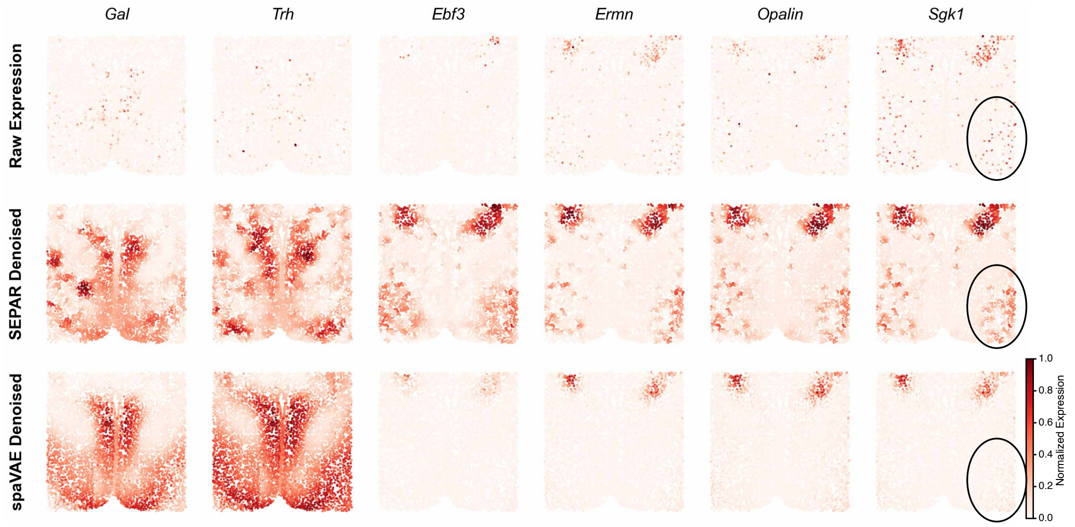

Supplementary Figure 21: Comparison of raw expression, SEPAR-denoised, and spaVAE-denoised expression for selected genes in the MERFISH dataset (n=5,926 cells). Expression values are normalized to [0,1] range within each panel for visualization. Black circles highlight that SEPAR preserves specific expression patterns.

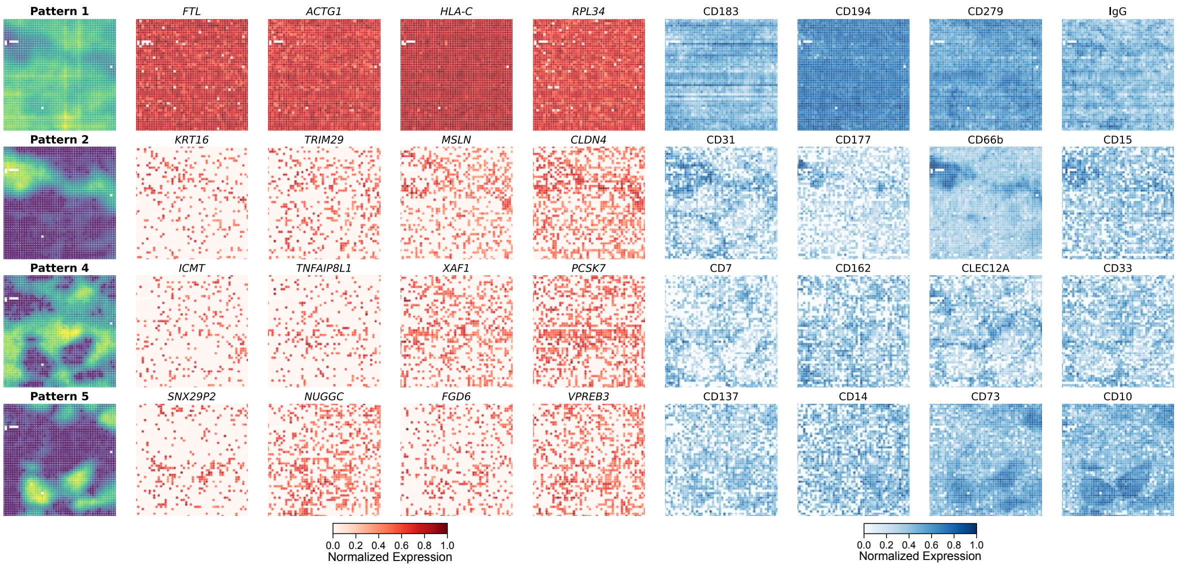

Supplementary Figure 22: Qualitative comparison of spatial expression between genes and proteins from the same pattern groups in spatial CITE-seq dataset (n=2,492 spots). Each row corresponds to Patterns 1, 2, 4, and 5, showing pattern distribution (column 1), selected genes (columns 2-5, red), and selected proteins (columns 6-9, blue) exhibiting coordinated spatial localization. Expression values are normalized to [0,1] range within each panel for visualization.

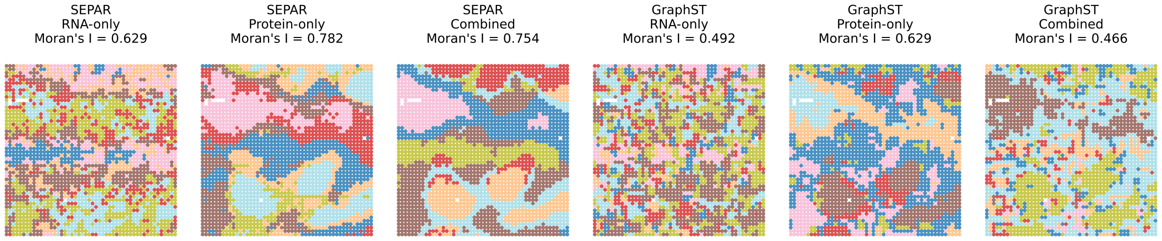

Supplementary Figure 23: Comparison of SEPAR and GraphST across individual and combined modalities on CITE-seq data (n=2,492 spots). Spatial clustering results show Moran's I values for RNA-only, protein-only, and combined RNA+protein analyses, demonstrating SEPAR's consistent advantages in spatial autocorrelation.

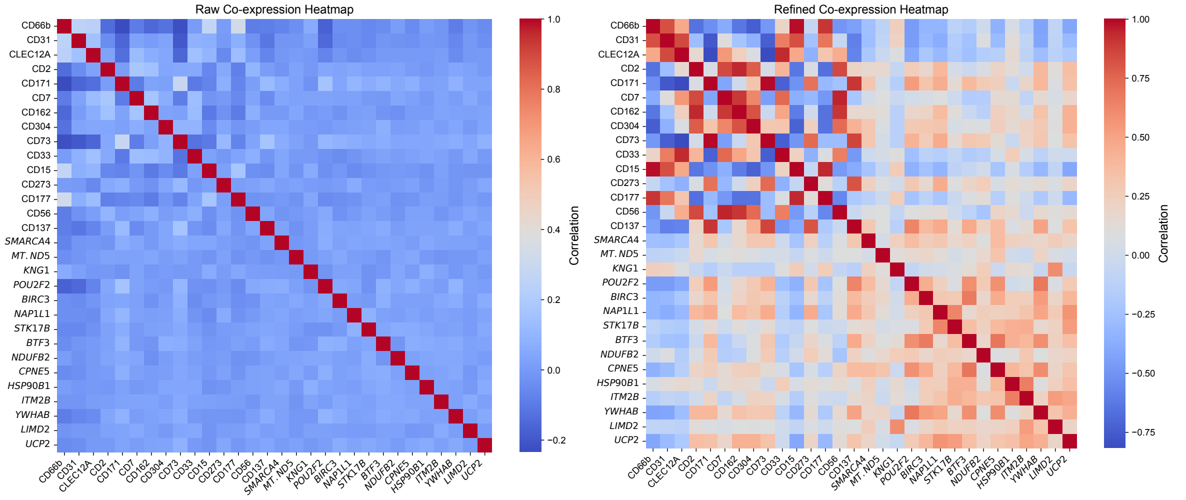

Supplementary Figure 24: Co-expression analysis within spatial CITE-seq dataset (15 genes and 15 proteins analyzed): (**a**) Heatmaps using raw expression data. (**b**) Heatmaps based on spatially refined expression.

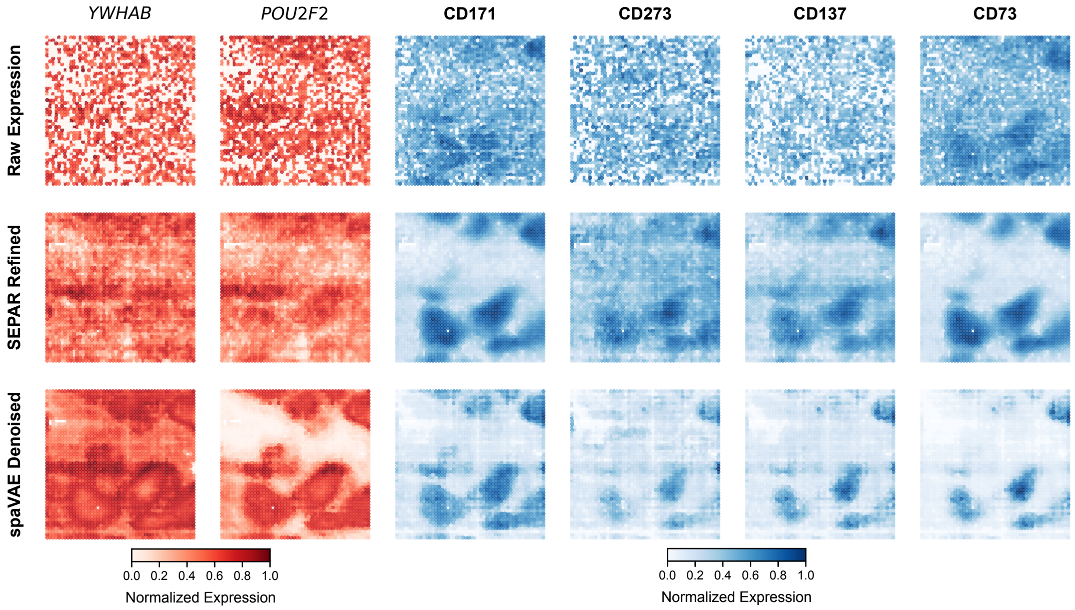

Supplementary Figure 25: Comparison of raw, SEPAR-refined, and spaVAE-denoised patterns in spatial CITE-seq dataset (n=2,492 spots). Expression values are normalized to [0,1] range within each panel for visualization.

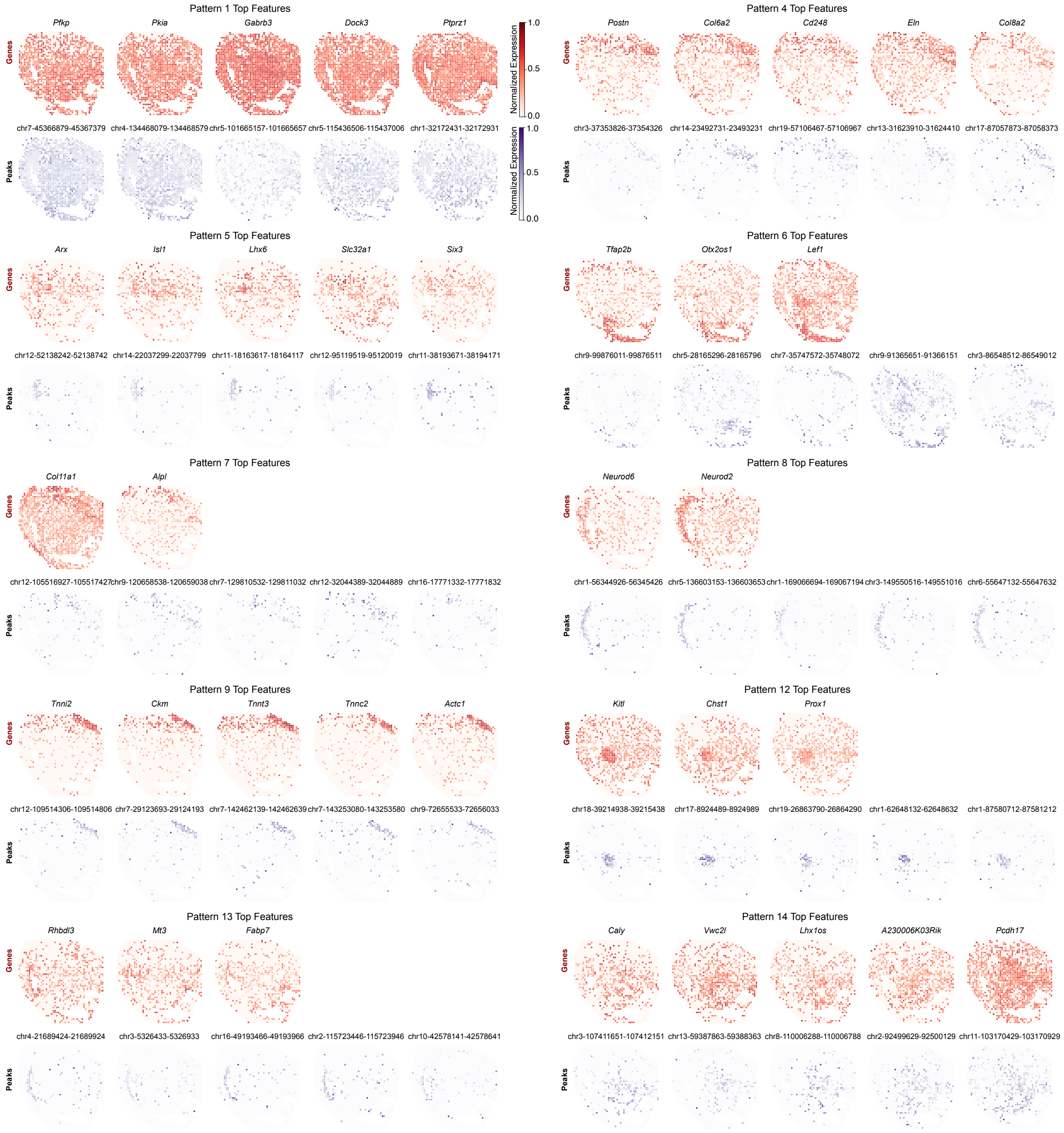

Supplementary Figure 26: Spatial visualization of top genes and peaks for selected MISAR-seq patterns from E15.5 mouse embryonic brain tissue (n=1,949 spots). Each panel shows spatial distribution of pattern-specific genes (red) and peaks (purple) for patterns 1, 4, 5, 6, 8, 9, 12, 13, and 14, demonstrating spatial coherence between chromatin accessibility and gene expression. Expression values are normalized to [0,1] range within each panel for visualization.

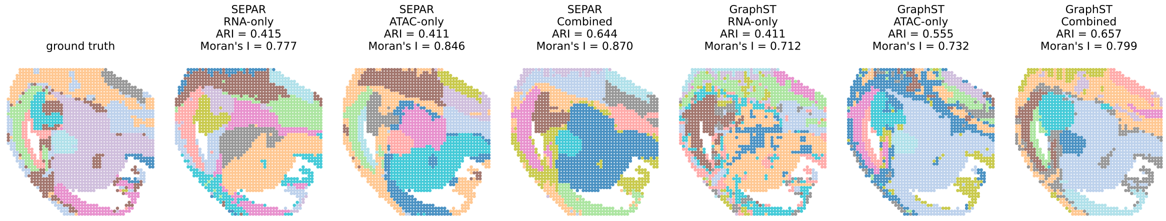

Supplementary Figure 27: Comprehensive comparison of spatial domain identification methods on MISAR-seq dataset (n=1,949 spots). Ground truth clustering compared with SEPAR's RNA-only, ATAC-only, and combined modalities, as well as GraphST results across different modalities. ARI values and Moran's I spatial autocorrelation scores are shown for quantitative evaluation.

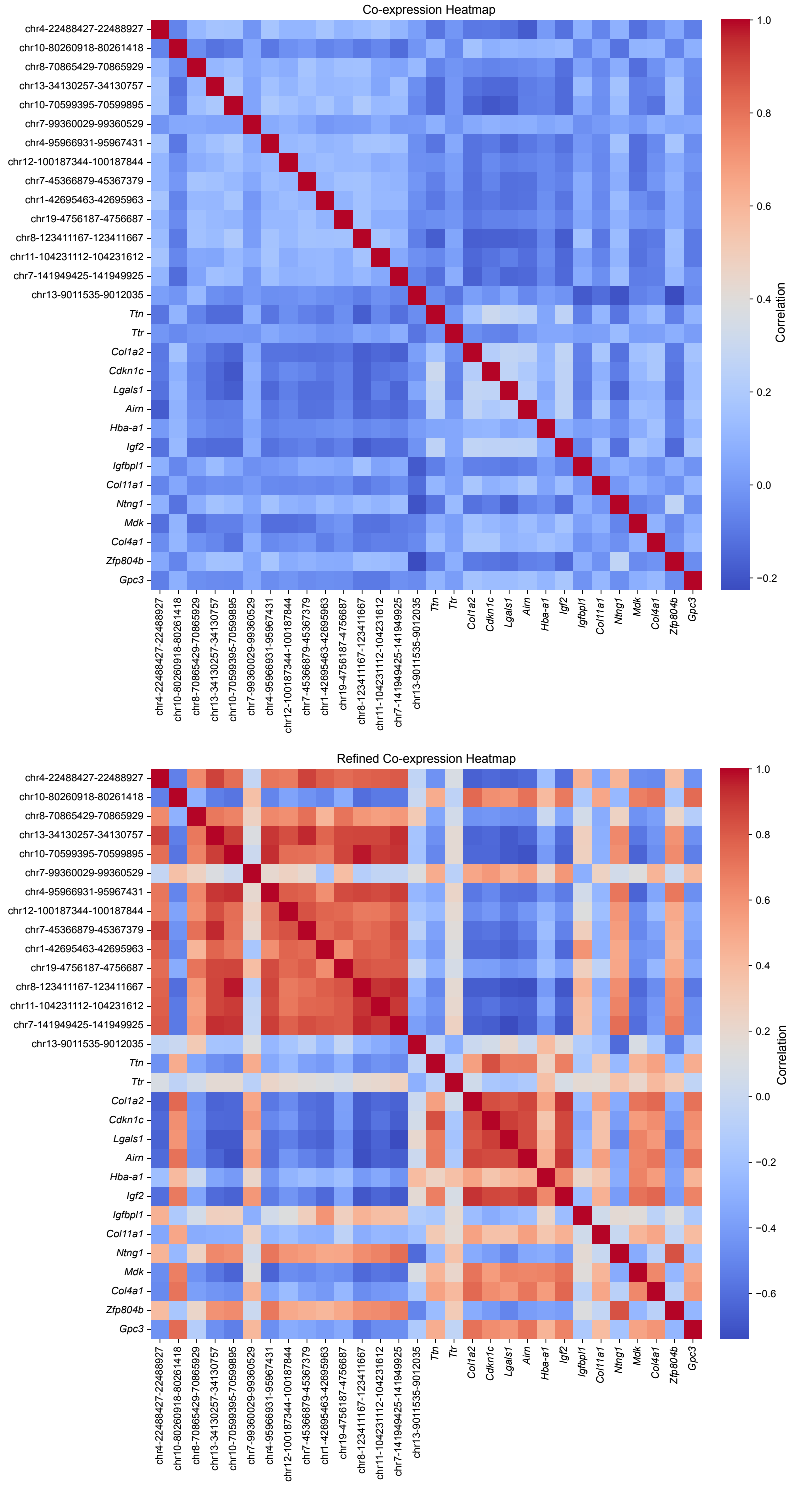

Supplementary Figure 28: Co-expression analysis within MISAR-seq dataset (15 genes and 15 peaks analyzed): (**a**) Heatmaps using raw expression. (**b**) Heatmaps based on spatially refined expression.

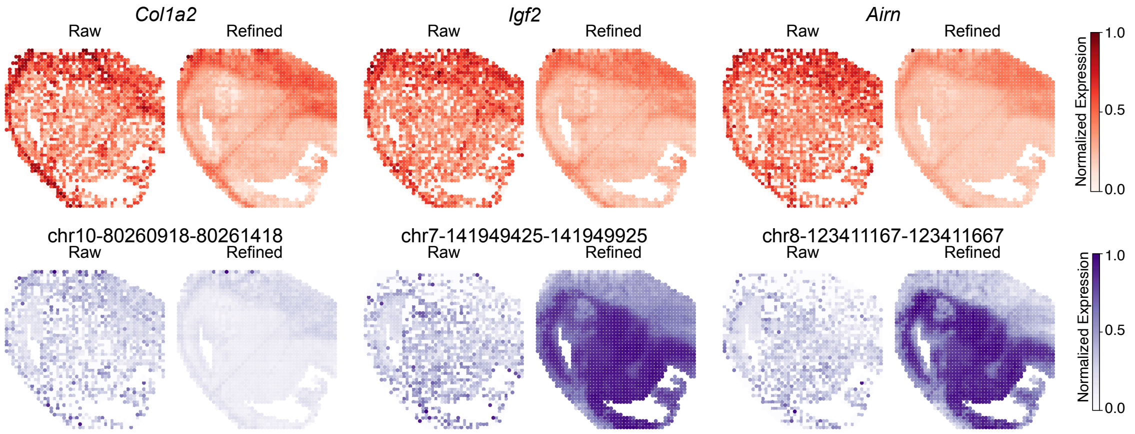

Supplementary Figure 29: Visualization of three genes and three peaks before and after refinement in MISAR-seq dataset (n=1,949 spots). Expression values are normalized to [0,1] range within each panel for visualization.

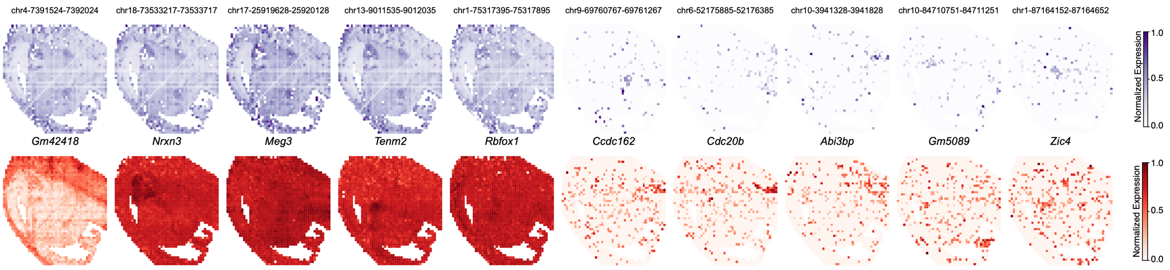

Supplementary Figure 30: SEPAR's identification of spatial and non-spatial variable peaks and genes in the MISAR-seq dataset (n=1,949 spots; 540 genes and 4,460 peaks analyzed). Expression values are normalized to [0,1] range within each panel for visualization.

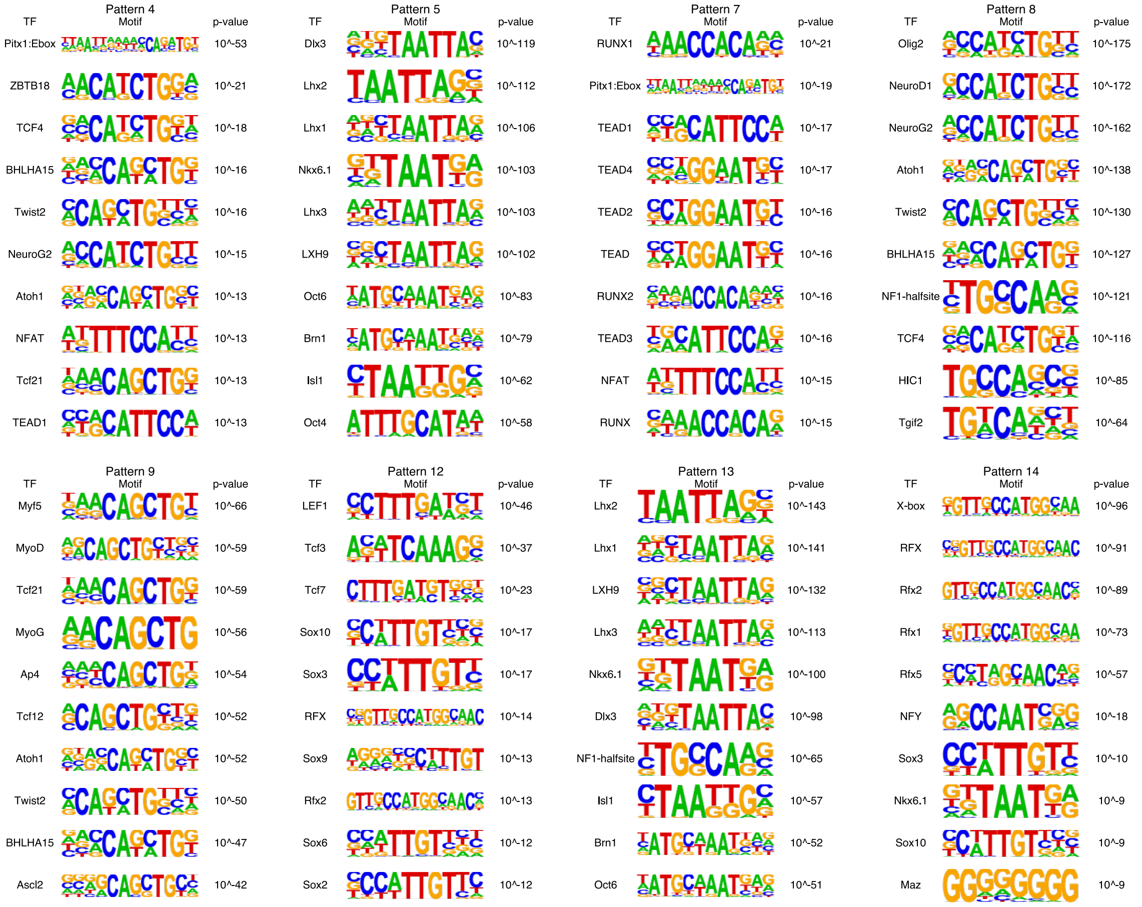

Supplementary Figure 31: Downstream motif enrichment analysis of pattern-specific peak sets identified by SEPAR from MISAR-seq data (E15.5 mouse embryonic brain; 8 peak sets from 1,949 spots). HOMER was used to identify enriched transcription factor binding motifs.

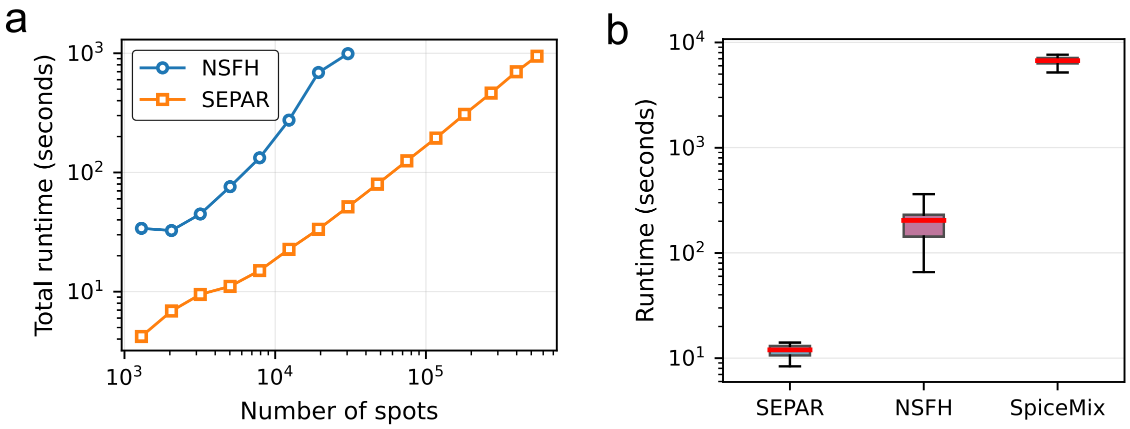

Supplementary Figure 32: **Computational performance and scalability analysis of SEPAR.** All experiments performed on Intel Xeon Gold 6226R (16 cores via SLURM) with NVIDIA A30 GPU. (**a**) Runtime scaling with dataset size using subsampled colorectal cancer VisiumHD data (ranging from 1,296 to 545,872 spots), demonstrating linear runtime growth and consistent computational efficiency. (**b**) Comparative runtime performance across NMF-based methods on 12 DLPFC slices. SEPAR shows over 10× faster performance than NSFH and over 100× faster than SpiceMix. Box plots show median (center line), quartiles (box boundaries), and whiskers extending to 1.5× IQR.

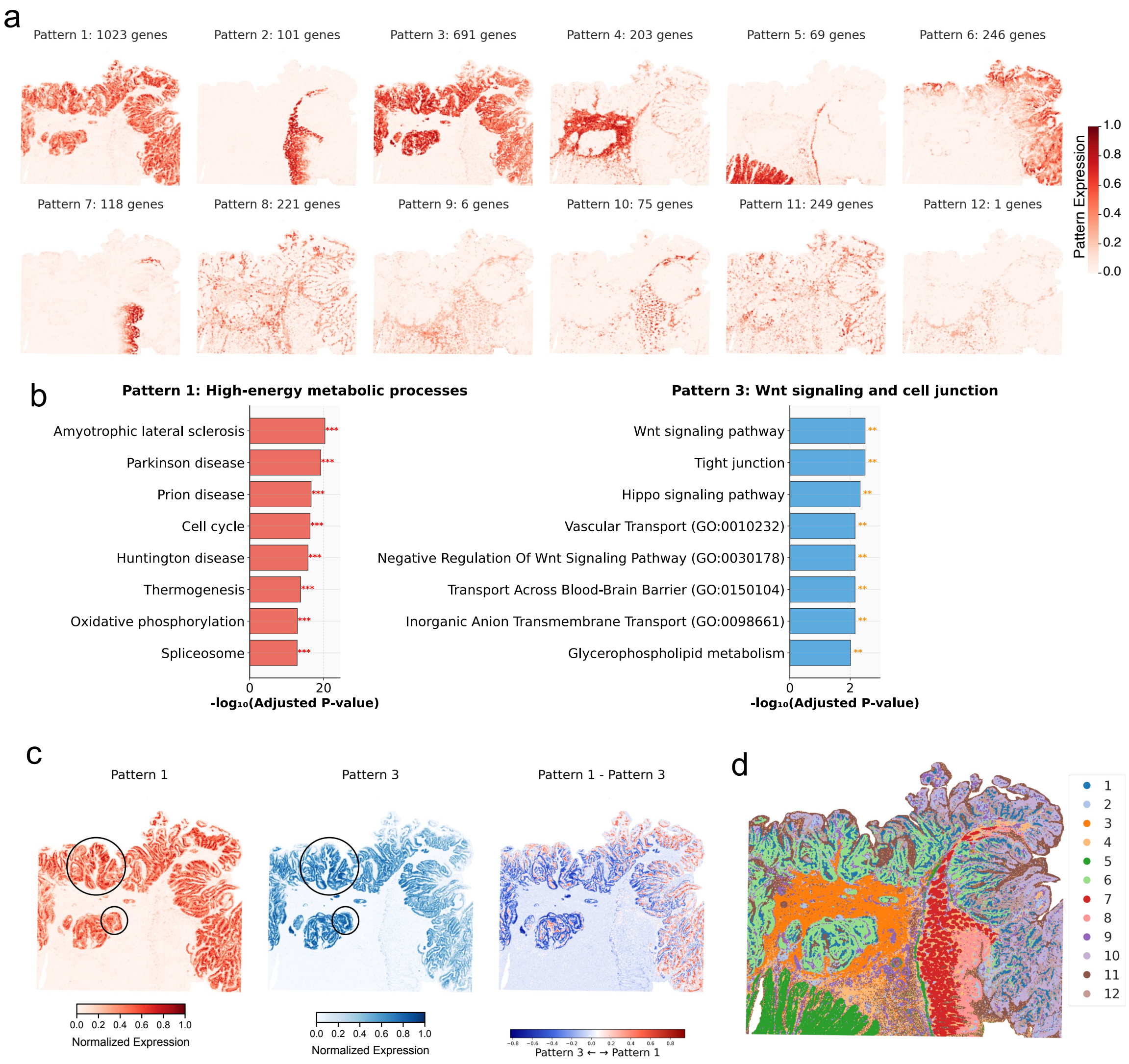

Supplementary Figure 33: **SEPAR analysis of colorectal cancer tissue spatial organization (VisiumHD, n=545,913 spots).** (**a**) Spatial distribution of 12 identified patterns. (**b**) Functional enrichment analysis of Pattern 1 and Pattern 3. (**c**) Spatial comparison and difference map of Pattern 1 and Pattern 3. (**d**) Spatial clustering results. Expression values are normalized to [0,1] range within each panel for visualization.

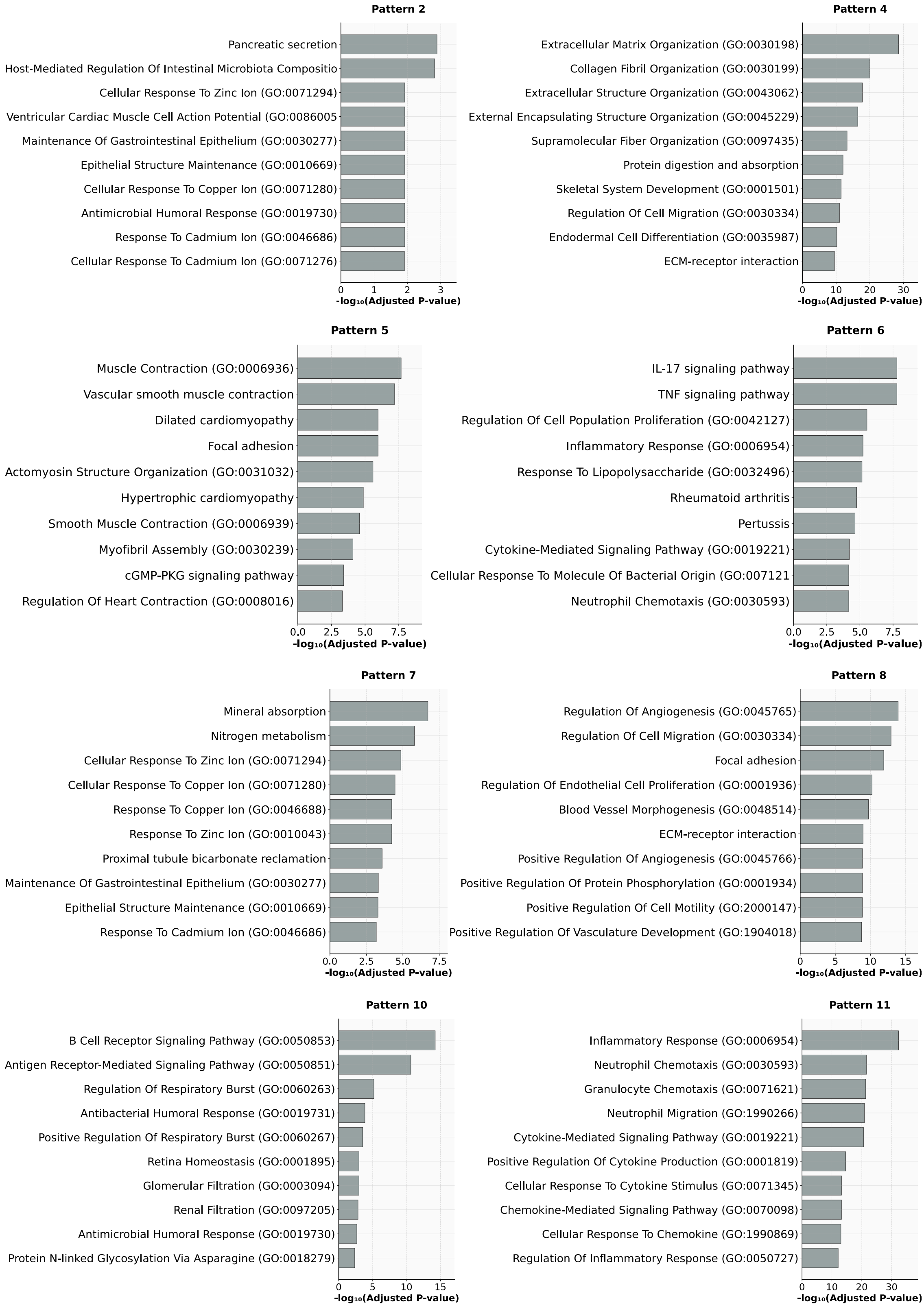

Supplementary Figure 34: Functional enrichment analysis of 8 pattern-specific gene sets in colorectal cancer tissue (gene set sizes: 6-246 genes).

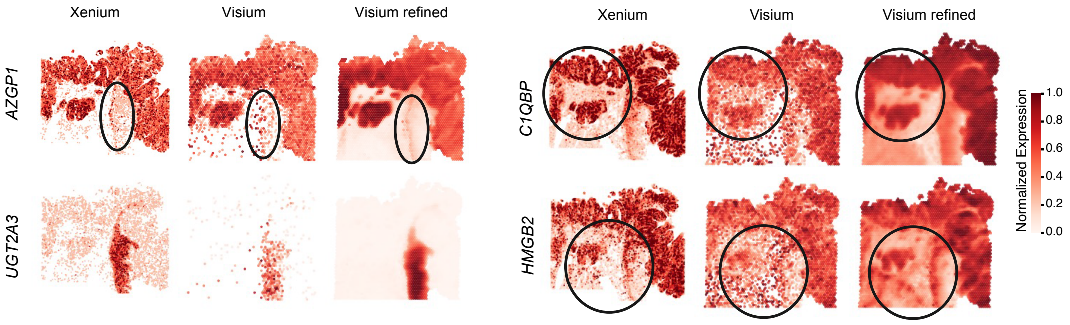

Supplementary Figure 35: Cross-technology validation using adjacent colorectal cancer tissue sections (Visium: n=4,269 spots; VisiumHD: n=545,913 spots; Xenium: n=340,837 cells). SEPAR-refined Visium expression (*Visium refined*) shows enhanced concordance with high-resolution Xenium data compared to original Visium data, as highlighted by black circles. Expression values are normalized to [0,1] range within each panel for visualization.

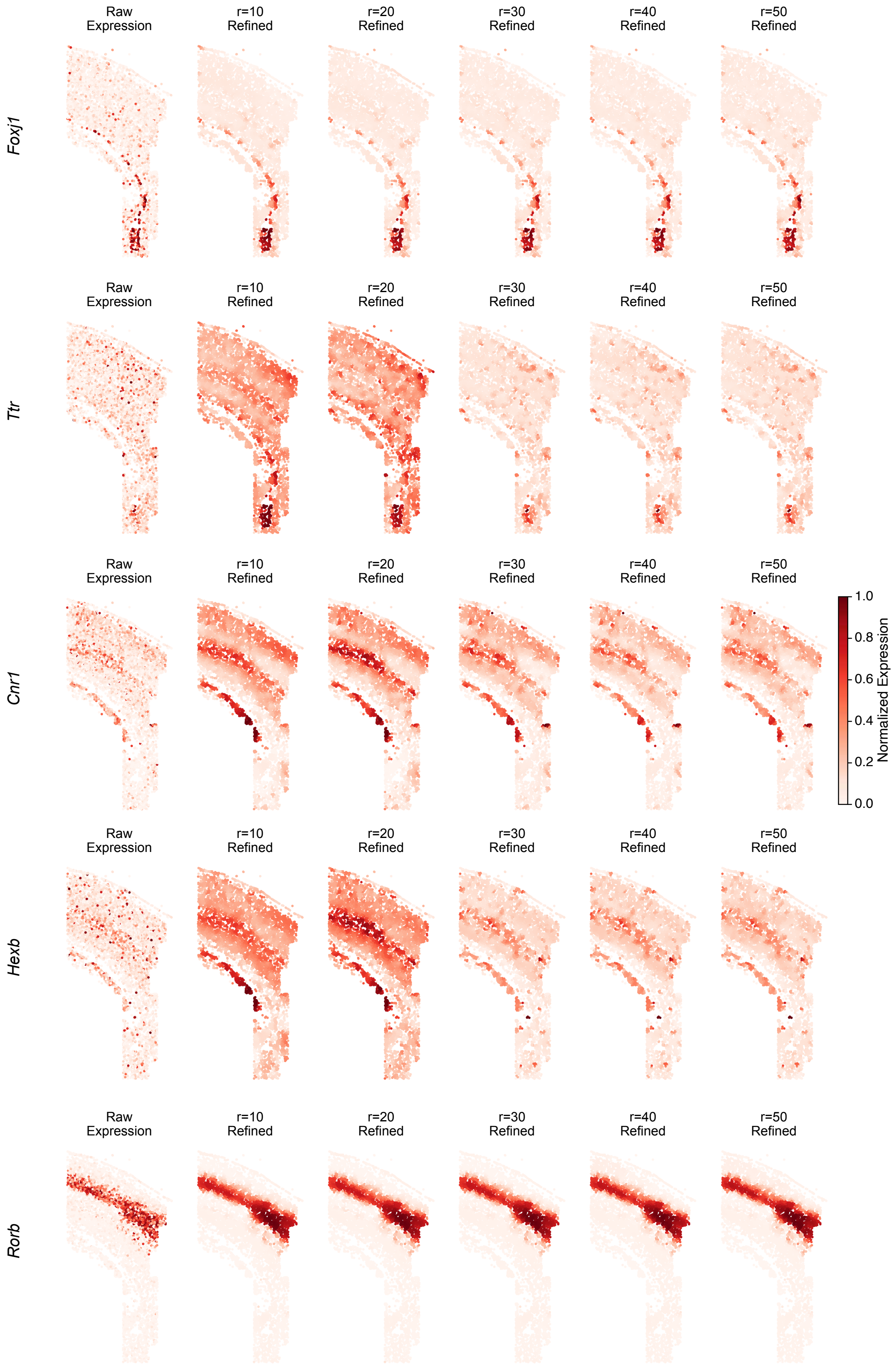

Supplementary Figure 36: Spatial expression refinement across r=10-50 on osmFISH dataset (n=4,839 cells). Expression values are normalized to [0,1] range within each panel for visualization. Increasing r weakens denoising and preserves finer details, with r∼20-30 providing practical balance.

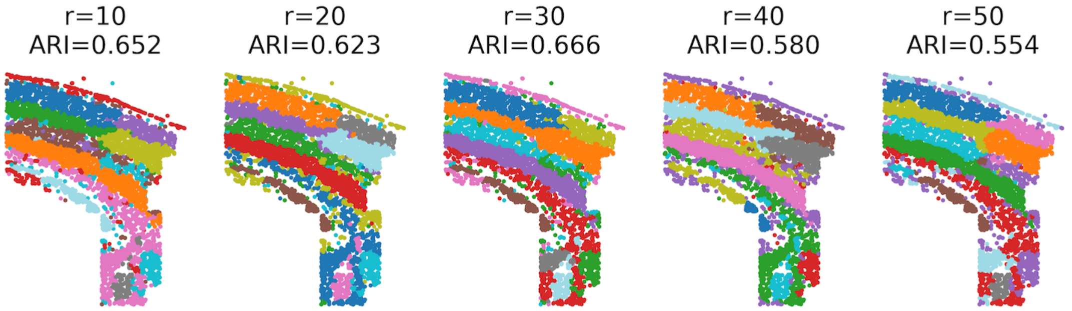

Supplementary Figure 37: Clustering results across different r values (10-50) on osmFISH dataset (n=4,839 cells) with corresponding ARI scores.

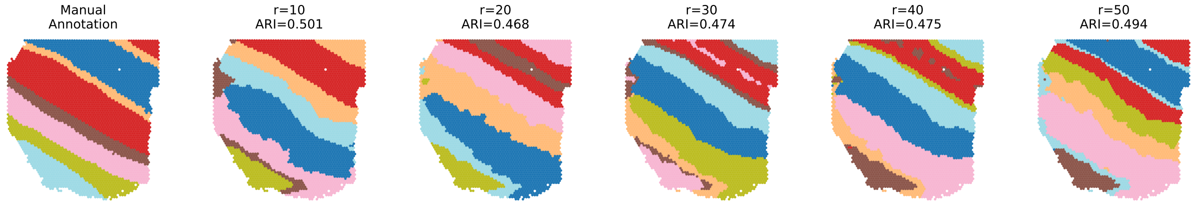

Supplementary Figure 38: Clustering results on DLPFC section 151507 (n=4,221 spots) across different r values (10-50) compared with manual annotation.

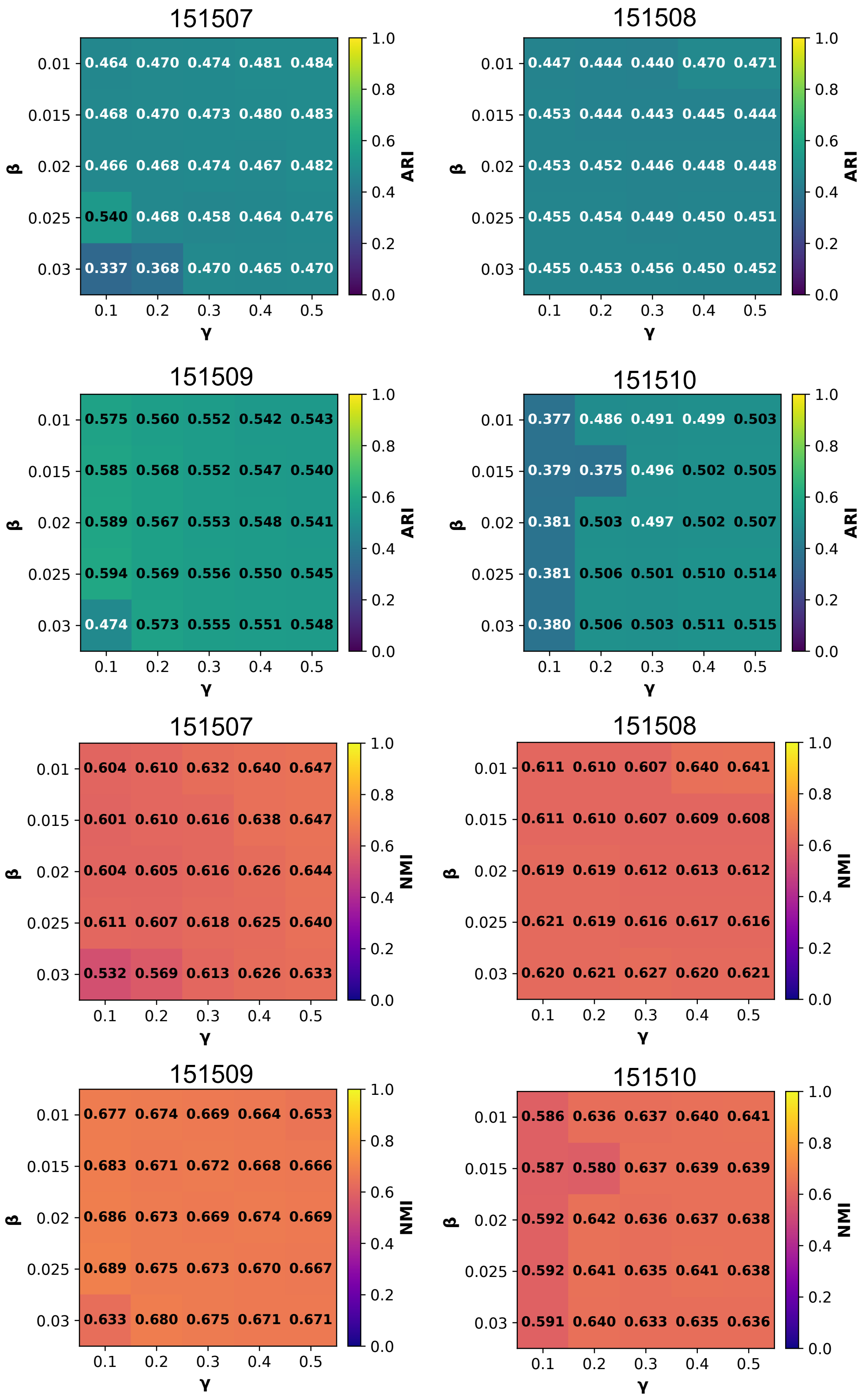

Supplementary Figure 39: Parameter sensitivity analysis across four DLPFC sections (151507-151510; n=4,221; 4,381; 4,788; 4,595 spots respectively). Heatmaps show ARI and NMI scores for different combinations of regularization parameters (β, γ) demonstrating stable clustering performance.

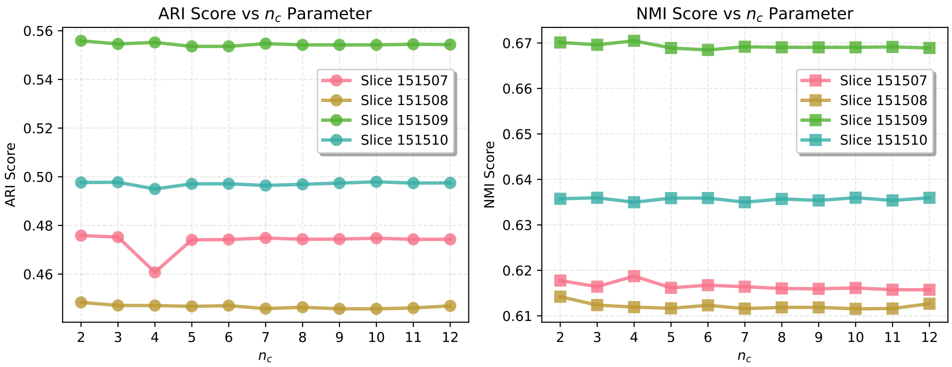

Supplementary Figure 40: Parameter sensitivity analysis for the number of clusters (n_c) on four DLPFC sections (151507-151510; n=4,221; 4,381; 4,788; 4,595 spots respectively). Both ARI and NMI scores remain stable across different n_c values (2-12), demonstrating robustness of SEPAR's clustering performance.

Supplementary References

References

[1] Michelli Faria de Oliveira, Juan Pablo Romero, Meii Chung, Stephen R Williams, Andrew D Gottscho, Anushka Gupta, Susan E Pilipauskas, Seayar Mohabbat, Nandhini Raman, David J Sukovich, et al. High-definition spatial transcriptomic profiling of immune cell populations in colorectal cancer. *Nature Genetics*, pages 1–12, 2025.

[2] Kok Hao Chen, Alistair N Boettiger, Jeffrey R Moffitt, Siyuan Wang, and Xiaowei Zhuang. Spatially resolved, highly multiplexed rna profiling in single cells. *Science*, 348(6233):aaa6090, 2015.

[3] Jeffrey R Moffitt, Dhananjay Bambah-Mukku, Stephen W Eichhorn, Eric Vaughn, Karthik Shekhar, Julio D Perez, Nimrod D Rubinstein, Junjie Hao, Aviv Regev, Catherine Dulac, et al. Molecular, spatial, and functional single-cell profiling of the hypothalamic preoptic region. *Science*, 362(6416):eaau5324, 2018.

[4] Zheng Li and Xiang Zhou. Bass: multi-scale and multi-sample analysis enables accurate cell type clustering and spatial domain detection in spatial transcriptomic studies. *Genome biology*, 23(1):168, 2022.

[5] Allen Institute for Brain Science. Allen reference atlas – mouse brain, 2011. Available from: `http://atlas.brain-map.org`.

[6] Tian Tian, Jie Zhang, Xiang Lin, Zhi Wei, and Hakon Hakonarson. Dependency-aware deep generative models for multitasking analysis of spatial omics data. *Nature Methods*, 21(8):1501–1513, 2024.

[7] Charles R Harris, K Jarrod Millman, Stéfan J Van Der Walt, Ralf Gommers, Pauli Virtanen, David Cournapeau, Eric Wieser, Julian Taylor, Sebastian Berg, Nathaniel J Smith, et al. Array programming with numpy. *Nature*, 585(7825):357–362, 2020.

[8] Pauli Virtanen, Ralf Gommers, Travis E Oliphant, Matt Haberland, Tyler Reddy, David Cournapeau, Evgeni Burovski, Pearu Peterson, Warren Weckesser, Jonathan Bright, et al. Scipy 1.0: fundamental algorithms for scientific computing in python. *Nature Methods*, 17(3):261–272, 2020.

[9] Fabian Pedregosa, Gaël Varoquaux, Alexandre Gramfort, Vincent Michel, Bertrand Thirion, Olivier Grisel, Mathieu Blondel, Peter Prettenhofer, Ron Weiss, Vincent Dubourg, et al. Scikit-learn: Machine learning in python. *the Journal of machine Learning research*, 12:2825–2830, 2011.

[10] Ryosuke Okuta, Yuya Unno, Daisuke Nishino, Shohei Hido, and Crissman Loomis. Cupy: A numpy-compatible library for nvidia gpu calculations. *Proceedings of Workshop on Machine Learning Systems (LearningSys) in The Thirty-first Annual Conference on Neural Information Processing Systems (NIPS)*, 6, 2017.

[11] Yunfei Hu, Manfei Xie, Yikang Li, Mingxing Rao, Wenjun Shen, Can Luo, Haoran Qin, Jihoon Baek, and Xin Maizie Zhou. Benchmarking clustering, alignment, and integration methods for spatial transcriptomics. *Genome Biology*, 25(1):212, 2024.

[12] Kristen R Maynard, Leonardo Collado-Torres, Lukas M Weber, Cedric Uytingco, Brianna K Barry, Stephen R Williams, Joseph L Catallini, Matthew N Tran, Zachary Besich, Madhavi Tippani, et al. Transcriptome-scale spatial gene expression in the human dorsolateral prefrontal cortex. *Nature neuroscience*, 24(3):425–436, 2021.
