# Supplementary material for: SEPAR enables spatial metagene discovery and associated molecular pattern characterization in spatial transcriptomics and multi-omics datasets
Source: Commun Biol. 2025 Dec 10;9:77. doi: 10.1038/s42003-025-09340-w (PMC12820152; doi:10.1038/s42003-025-09340-w)
Supplement: Supplementary file 2 — Description of Additional Supplementary files [file 42003_2025_9340_MOESM2_ESM.pdf]

Description of Additional Supplementary files

File name: Supplementary Data 1

Description: Supplementary Data 1 includes all the computational results for Figures 2-7.
