# Supplementary material for: SEPAR enables spatial metagene discovery and associated molecular pattern characterization in spatial transcriptomics and multi-omics datasets
Source: Commun Biol. 2025 Dec 10;9:77. doi: 10.1038/s42003-025-09340-w (PMC12820152; doi:10.1038/s42003-025-09340-w)
Supplement: Supplementary file 4 — Article File-Pdf [file 42003_2025_9340_MOESM4_ESM.pdf]

nature portfolio

Corresponding author(s): DBPR COMMSBIO-25-2937A

Last updated by author(s): Nov 9, 2025

Reporting Summary

Nature Portfolio wishes to improve the reproducibility of the work that we publish. This form provides structure for consistency and transparency in reporting. For further information on Nature Portfolio policies, see our Editorial Policies and the Editorial Policy Checklist.

Statistics

For all statistical analyses, confirm that the following items are present in the figure legend, table legend, main text, or Methods section.

n/a	Confirmed	
☐	☒	The exact sample size (*n*) for each experimental group/condition, given as a discrete number and unit of measurement
☒	☐	A statement on whether measurements were taken from distinct samples or whether the same sample was measured repeatedly
☐	☒	The statistical test(s) used AND whether they are one- or two-sided<br>*Only common tests should be described solely by name; describe more complex techniques in the Methods section.*
☒	☐	A description of all covariates tested
☒	☐	A description of any assumptions or corrections, such as tests of normality and adjustment for multiple comparisons
☐	☒	A full description of the statistical parameters including central tendency (e.g. means) or other basic estimates (e.g. regression coefficient) AND variation (e.g. standard deviation) or associated estimates of uncertainty (e.g. confidence intervals)
☐	☒	For null hypothesis testing, the test statistic (e.g. F, t, r) with confidence intervals, effect sizes, degrees of freedom and P value noted<br>*Give P values as exact values whenever suitable.*
☒	☐	For Bayesian analysis, information on the choice of priors and Markov chain Monte Carlo settings
☒	☐	For hierarchical and complex designs, identification of the appropriate level for tests and full reporting of outcomes
☐	☒	Estimates of effect sizes (e.g. Cohen's *d*, Pearson's *r*), indicating how they were calculated

Our web collection on statistics for biologists contains articles on many of the points above.

Software and code

Policy information about availability of computer code

Data collection	All data analyzed within this manuscript are publicly available. No additional software was used for the data collection process.
Data analysis	We performed the data analysis using our developed SEPAR software in this paper, which is available at GitHub repository: [https://github.com/zerovain/SEPAR]. The following software packages were used in our code: pandas V2.0.3, numpy V1.23.5, scanpy V1.9.6, anndata V0.8.0, matplotlib V3.6.1, scipy V1.10.0, scikit-learn V1.2.0, tqdm V4.64.1, cupy V10.6.0.

For manuscripts utilizing custom algorithms or software that are central to the research but not yet described in published literature, software must be made available to editors and reviewers. We strongly encourage code deposition in a community repository (e.g. GitHub). See the Nature Portfolio guidelines for submitting code & software for further information.

Data

Policy information about availability of data

All manuscripts must include a data availability statement. This statement should provide the following information, where applicable:

- Accession codes, unique identifiers, or web links for publicly available datasets
- A description of any restrictions on data availability
- For clinical datasets or third party data, please ensure that the statement adheres to our policy

All data analyzed in this paper are available in raw form from their original authors. Specifically, the human DLPFC dataset can be accessed at spatialLIBD project website, (http://research.libd.org/spatialLIBD/); the processed mouse olfactory bulb Stereoseq data is available at https://doi.org/10.5281/zenodo.8356092; the

MERFISH data can be downloaded from https://doi.org/10.5061/dryad.8t8s248; the osmFISH data is available at http://linnarssonlab.org/osmFISH/availability/; the spatial CITE-seq is accessible on https://www.ncbi.nlm.nih.gov/geo/query/acc.cgi?acc=GSE213264 and MISAR-seq datasets are available at https://doi.org/10.5281/zenodo.7480069. The human colorectal cancer (CRC) datasets including VisiumHD, Visium, and Xenium data for P2CRC samples are available at the following website: https://www. 10xgenomics.com/products/visium-hd-spatial-gene-expression/dataset-human-crc.

Research involving human participants, their data, or biological material

Policy information about studies with human participants or human data. See also policy information about sex, gender (identity/presentation), and sexual orientation and race, ethnicity and racism.

Reporting on sex and gender	N/A
Reporting on race, ethnicity, or other socially relevant groupings	N/A
Population characteristics	N/A
Recruitment	N/A
Ethics oversight	N/A

Note that full information on the approval of the study protocol must also be provided in the manuscript.

Field-specific reporting

Please select the one below that is the best fit for your research. If you are not sure, read the appropriate sections before making your selection.

☒ Life sciences ☐ Behavioural & social sciences ☐ Ecological, evolutionary & environmental sciences

For a reference copy of the document with all sections, see nature.com/documents/nr-reporting-summary-flat.pdf

Life sciences study design

All studies must disclose on these points even when the disclosure is negative.

Sample size	We used publicly available dataset in this paper and no sample size calculation was performed.
Data exclusions	We performed quality control of spatial omics data based on the common used and pre-established criteria in this field.
Replication	N/A
Randomization	N/A
Blinding	N/A

Reporting for specific materials, systems and methods

We require information from authors about some types of materials, experimental systems and methods used in many studies. Here, indicate whether each material, system or method listed is relevant to your study. If you are not sure if a list item applies to your research, read the appropriate section before selecting a response.

Materials & experimental systems

n/a	Involved in the study	
☒	☐	Antibodies
☒	☐	Eukaryotic cell lines
☒	☐	Palaeontology and archaeology
☒	☐	Animals and other organisms
☒	☐	Clinical data
☒	☐	Dual use research of concern
☒	☐	Plants

Methods

n/a	Involved in the study	
☒	☐	ChIP-seq
☒	☐	Flow cytometry
☒	☐	MRI-based neuroimaging

Plants

Seed stocks	N/A
Novel plant genotypes	N/A
Authentication	N/A
